# circPLIN2 promotes clear cell renal cell carcinoma progression by binding IGF2BP proteins and miR-199a-3p

**DOI:** 10.1038/s41419-022-05488-z

**Published:** 2022-12-09

**Authors:** Bin Zhao, Cong Huang, Jie Pan, Hao Hu, Xiaojuan Liu, Kaoyuan Zhang, Fenli Zhou, Xin Shi, Jun Wu, Bo Yu, Xiaofan Chen, Wei Zhang

**Affiliations:** 1 Biomedical Research Institute, Shenzhen Peking University - The Hong Kong University of Science and Technology Medical Center, Shenzhen, Guangdong Province China; 2 Department of Dermatology, Skin Research Institute of Peking University Shenzhen Hospital, Peking University Shenzhen Hospital, Shenzhen Peking University - The Hong Kong University of Science and Technology Medical Center, Shenzhen, Guangdong Province China; 3 Department of Pathology, Stanford University School of Medicine, Palo Alto, CA USA; 4 Department of Neurology, Peking University Shenzhen Hospital, Shenzhen, Guangdong Province China; 5 Greater Bay Biomedical Innovation Center, Shenzhen Bay Laboratory, Shenzhen, Guangdong Province China

**Keywords:** Renal cell carcinoma, Targeted therapies, RNA

## Abstract

Recent evidence has indicated that circular RNAs (circRNAs), a novel type of regulatory RNA, play important roles in the development and progression of various cancers. However, the potential regulatory roles and molecular mechanisms of circRNAs in clear cell renal cell carcinoma (ccRCC) remain largely unclear. Here, we explored circRNA expression profiles in 10 paired samples of RCC (including cancer tissues and surrounding tissues) from the Gene Expression Omnibus (GEO) datasets GSE124453 and GSE108735. We initially identified hsa_circ_0086457, designated circPLIN2, derived from exons 4 to 5 of the PLIN2 gene. We observed that circPLIN2 was preferentially located in the cytoplasm and was more stable than its linear counterpart PLIN2. circPLIN2 was significantly upregulated in ccRCC cells and tissues, and its overexpression was correlated with higher clinical stage and worse prognosis for ccRCC patients. Moreover, gain- and loss-of-function assays indicated that circPLIN2 promoted ccRCC cell proliferation, migration, and invasion in vitro and ccRCC tumor growth and metastasis in vivo. Mechanistically, circPLIN2 not only increased the stability of the c-Myc and MARCKSL1 mRNAs by binding to the KH domains of IGF2BP proteins but also competitively sponged miR-199a-3p to abolish the repressive effect of miR-199a-3p on ZEB1 expression, which ultimately resulted in ccRCC tumorigenesis and progression. Collectively, our results suggest that circPLIN2 may represent a promising diagnostic and prognostic biomarker and a potential therapeutic target for ccRCC patients.

## Introduction

Renal cell carcinoma (RCC) is one of the most common malignant tumors in humans, and its high morbidity and mortality rates, with 73,750 new cases and 14,830 deaths estimated in 2020 in the US, make it a growing global health problem [1]. Clear cell renal cell carcinoma (ccRCC) is the most common type of RCC, accounting for approximately 70–75% of RCC cases [2]. Currently, the gold standard for the diagnosis and treatment of ccRCC patients is the early detection of microtumor lesions and radical surgical resection of localized ccRCC, which generally result in excellent long-term disease-free survival (DFS) [3, 4]. However, the prognosis of patients with advanced ccRCC is poor due to local tumor recurrence or distant metastasis, even after radical nephrectomy [5]. In addition, the majority of ccRCCs are resistant to both traditional chemotherapy and radiotherapy once they recur or metastasize, leading to shorter overall survival for patients with advanced ccRCC [6, 7]. Therefore, studies elucidating the potential mechanisms underlying the pathogenesis of ccRCC and identifying new effective therapeutic approaches for ccRCC are urgently needed.

Recently, circular RNAs (circRNAs) have been characterized as covalently closed loop structures without a 5′ cap and a 3′ poly(A) tail that are formed by back-splicing events and have attracted the attention of many researchers [8–10]. circRNAs are widely expressed in a variety of eukaryotes and have greater stability and a stronger resistance to digestion by RNase R treatment than their linear counterpart mRNAs [8–10]. In addition, circRNAs have many important regulatory functions. For instance, circRNAs function as competing endogenous RNAs (ceRNAs) to sponge miRNAs to regulate the expression of downstream genes [11–14] and interact with RNA-binding proteins to regulate protein functions [15–20]. In recent years, accumulating evidence has shown that circRNAs encode functional microproteins by the cap-independent translation pathway [21–23] or m^6^A (N^6^-methyladenosine) modification [24–26]. circRNAs, a novel type of regulatory RNA molecule, play important roles in the development and progression of various cancers [27–31]. Meanwhile, the conserved, stable, and specific spatiotemporal characteristics of circRNAs make them excellent biomarkers for tumor diagnosis and prognosis and potential therapeutic targets for malignant tumors [32–34]. However, to date, the key regulatory roles and underlying molecular mechanisms of circRNAs in the development and progression of ccRCC remain largely unclear.

In this study, we investigated circRNA expression profiles in 10 paired samples of RCC (including cancer tissues and surrounding tissues) from the GEO datasets GSE124453 and GSE108735. We initially identified hsa_circ_0086457, termed circPLIN2, derived from exons 4 to 5 of the PLIN2 gene. circPLIN2 was preferentially located in the cytoplasm and was more stable than its linear transcript PLIN2. circPLIN2 was markedly upregulated in ccRCC cells and tissues, and its overexpression was correlated with higher clinical stage and worse prognosis for ccRCC patients. Gain- and loss-of-function assays indicated that circPLIN2 promoted ccRCC cell proliferation, migration, and invasion in vitro and ccRCC tumor growth and metastasis in vivo. Mechanistically, circPLIN2 not only increased the stability of the c-Myc and MARCKSL1 mRNAs by binding to the KH domains of IGF2BP proteins but also competitively sponged miR-199a-3p to abolish the repressive effect of miR-199a-3p on ZEB1 expression, which ultimately resulted in ccRCC tumorigenesis and progression.

## Results

### Identification and characteristics of circPLIN2 in ccRCC

**Fig. 1:**
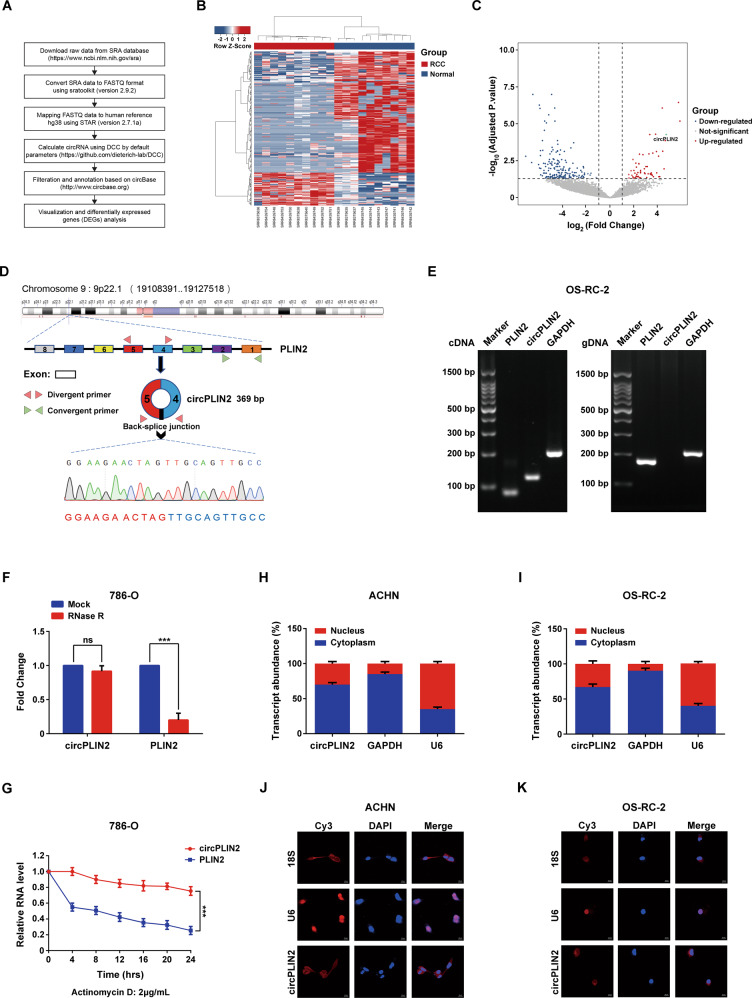
Identification and characteristics of circPLIN2 in ccRCC. **A** The flowchart delineates the steps for exploring circRNA expression profiling in 10 paired samples of RCC by meta-analysis of the GSE124453 and GSE108735 datasets from GEO. **B** Heatmap of circRNA expression in 10 paired samples of RCC. **C** Volcano plot of differentially expressed circRNAs in 10 paired samples of RCC. **D** Genomic localization of circPLIN2. circPLIN2 is derived from exons 4 to 5 of the parental PLIN2 gene and has a length of 369 nucleotides. The back-splice junction of circPLIN2 was identified by Sanger sequencing. Divergent primer for circPLIN2 and convergent primer for PLIN2. **E** PCR and agarose gel electrophoresis analysis of circPLIN2 and its linear isoform PLIN2 in the cDNA and gDNA obtained from OS-RC-2 cells. cDNA, complementary DNA obtained after reverse transcription of RNA; gDNA, genomic DNA. circPLIN2, 128 bp; PLIN2, 90 bp; GAPDH, 197 bp. bp, base pair. GAPDH served as a positive control. **F** RT–qPCR analysis of the abundance of circPLIN2 and PLIN2 in 786-O cells treated with RNase R. **G** RT–qPCR analysis of the levels of circPLIN2 and PLIN2 in 786-O cells treated with actinomycin D (2 μg/mL) at the indicated time points. **H**, **I** RT–qPCR analysis of the abundance of circPLIN2 in the nuclear and cytoplasmic fractions of ACHN (**H**) and OS-RC-2 (**I**) cells. GAPDH served as a positive cytoplasmic control, and U6 served as a positive nuclear control. **J**, **K** Fluorescence in situ hybridization (FISH) analysis of circPLIN2 levels in the nuclear and cytoplasmic fractions of ACHN (**J**) and OS-RC-2 (**K**) cells. All probes are labeled with Cy3. 18 S was used as a positive cytoplasmic control, and U6 was used as a positive nuclear control. Two-tailed Student’s t test. The error bars represent S.D. ns, not significant; ***p < 0.001.

We first analyzed the expression profiles of circRNAs in human ccRCC to explore the regulatory roles of circRNAs and their underlying molecular mechanisms in the development and progression of human ccRCC. We performed a joint analysis of the circRNA expression data for 10 paired samples of RCC (including cancer tissues and surrounding tissues) from the GEO datasets GSE124453 and GSE108735 (http://www.ncbi.nlm.nih.gov/geo) (Fig. 1A and Supplementary Table 1). A total of 12,299 circRNAs were identified (Fig. 1B and Supplementary Table 2). Among all circRNAs, 243 were identified as differentially expressed circRNAs between RCC and normal tissues, including 186 downregulated circRNAs and 57 upregulated circRNAs in RCC (Fig. 1C and Supplementary Table 2). We found that hsa_circ_0086457, designated circPLIN2, was significantly upregulated in RCC samples (Fig. 1C).

circPLIN2 is a circular RNA molecule derived from exons 4 to 5 of the PLIN2 gene on human chromosome 9 (9p22.1) with a length of 369 nucleotides (Fig. 1D). The back-splice junction of circPLIN2 was amplified using divergent primers and confirmed by Sanger sequencing, and the result was consistent with the circBase database annotation (http://www.circbase.org) (Fig. 1D). Subsequently, PCR amplification and agarose gel electrophoresis using divergent and convergent primers further revealed that circPLIN2 was amplified from cDNA templates but not gDNA templates compared to PLIN2 and GAPDH (Fig. 1E), consistent with the general characteristics of circRNAs. We next investigated the resistance of circPLIN2 to RNase R digestion, and the results indicated that circPLIN2 was more tolerant to RNase R digestion than its linear counterpart PLIN2 (Fig. 1F). In addition, actinomycin D, an inhibitor of transcription, was applied to determine the half-life of circPLIN2 in ccRCC cells, and the content of circPLIN2 decreased slowly over time compared with the linear PLIN2 transcript in 786-O cells cultured in the presence of 2 μg/mL actinomycin D, suggesting that circPLIN2 was more stable or had a longer half-life than its linear counterpart PLIN2 (Fig. 1G). We performed RT–qPCR analysis to determine the abundance of nuclear and cytoplasmic circPLIN2 in ccRCC cells. Notably, circPLIN2 was preferentially located in the cytoplasm of ACHN (Fig. 1H) and OS-RC-2 (Fig. 1I) cells, consistent with the results of the fluorescence in situ hybridization (FISH) assays (Fig. 1J, K). Overall, circPLIN2, the back-spliced product of the parent gene PLIN2, was preferentially distributed in the cytoplasm of ccRCC cells and had a longer half-life and a stronger resistance to RNase R digestion than its linear counterpart PLIN2.

### circPLIN2 is significantly upregulated in ccRCC and correlates with disease progression and the poor prognosis of ccRCC patients

**Fig. 2:**
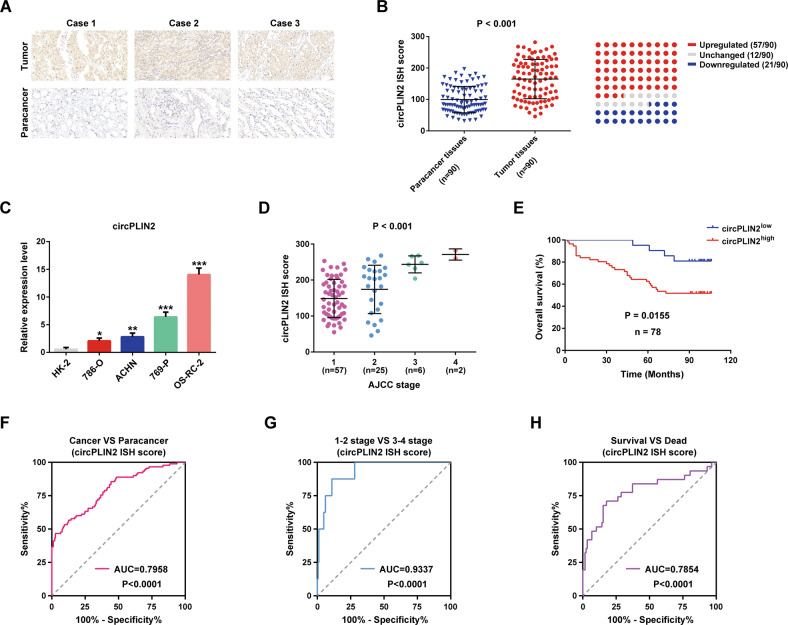
circPLIN2 is significantly upregulated in ccRCC and correlated with the progression and poor prognosis of ccRCC patients. **A** Representative images of in situ hybridization (ISH) for circPLIN2 expression in three pairs of samples from the ccRCC tissue microarray. Scale bar, 20 μm. **B** circPLIN2 ISH staining scores in 90 pairs of cancerous and paracancerous tissues are shown on the left, and the expression profiles of circPLIN2 in 90 patients with ccRCC are shown on the right. **C** RT–qPCR analysis of the relative expression levels of circPLIN2 in a panel of four human ccRCC cell lines (786-O, ACHN, 769-P, and OS-RC-2) and an immortalized proximal tubule epithelial cell line (HK-2). The relative circPLIN2 expression level was normalized to GAPDH. **D** circPLIN2 ISH staining scores in ccRCC tissues (*n* = 90) in different AJCC stages. **E** Overall survival curve of ccRCC patients with high (*n* = 57) or low (*n* = 21) circPLIN2 expression. Statistical significance was determined using the Kaplan–Meier test. **F**–**H** The receiver operating characteristic curve (ROC) analysis for cancer and paracancer (**F**), AJCC stage 1–2 and 3–4 (**G**), survival and death (**H**) in 90 ccRCC patients based on the circPLIN2 ISH staining scores. Two-tailed Student’s t test. The error bars represent S.D. *p < 0.05, **p < 0.01, and ***p < 0.001.

Furthermore, in situ hybridization staining was performed on a tissue microarray of human ccRCC, including 90 cases of tumor tissues and adjacent tissues, with probes specific for circPLIN2 to validate its expression. Three representative cases of in situ hybridization staining for circPLIN2 expression in the tissue microarray were shown (Fig. 2A). We found that circPLIN2 was significantly upregulated in ccRCC tissues compared with surrounding normal tissues (Fig. 2B, left panel), accounting for approximately 63% (57/90) of 90 ccRCC specimens (Fig. 2B, right panel). To further examine circPLIN2 overexpression in ccRCC, we used a panel of four human ccRCC cell lines (786-O, ACHN, 769-P, and OS-RC-2 cells) and HK-2 cells (a proximal tubule epithelial cell line) to test circPLIN2 expression by RT–qPCR. The results showed that circPLIN2 was observably overexpressed in ccRCC cells compared to HK-2 cells (Fig. 2C), consistent with the results of in situ hybridization staining assays (Fig. 2A, B).

Furthermore, substantially higher circPLIN2 levels were detected in ccRCC tissues with advanced American Joint Committee on Cancer (AJCC) stages (AJCC stage 3–4) than in ccRCC tissues with early AJCC stages (AJCC stage 1–2) (Fig. 2D). In addition, we analyzed the correlation between circPLIN2 expression and clinicopathological characteristics in 90 ccRCC patients. The results showed that circPLIN2 expression was only significantly correlated with tumor differentiation, and the higher the expression level of circPLIN2, the worse the tumor differentiation and the higher the malignant grade of the tumor (Table 1). The survival curve analysis showed that ccRCC patients with high circPLIN2 expression had a markedly lower overall survival rate than ccRCC patients with low circPLIN2 expression (Fig. 2E). Moreover, the univariate Cox proportional hazard regression analysis showed that the differential expression of circPLIN2 was significantly correlated with the overall survival in 78 ccRCC patients (*P* = 0.026) (Table 2), consistent with the results of the Kaplan–Meier analysis (Fig. 2E). However, the multivariate Cox proportional hazard regression analysis showed that the differential expression of circPLIN2 was not associated with the overall survival in 78 ccRCC patients (*P* = 0.206) (Table 2), which may be explained by the small number of patients involved or the presence of some factors that interfered with the true results. The receiver operating characteristic (ROC) curve indicated that circPLIN2 expression showed excellent diagnostic performance for cancer and paracancer (Fig. 2F), AJCC stage 1–2 and stage 3–4 (Fig. 2G), and the survival and death of ccRCC patients (Fig. 2H). Collectively, these results suggested that circPLIN2 was significantly upregulated in ccRCC cells and tissues and that its overexpression was correlated with higher clinical stage and worse prognosis for ccRCC patients.

**Table 1:** The relationship between circPLIN2 expression and the clinicopathological characteristics in 90 ccRCC patients.

Variables	circPLIN2 expression			*P* value
	High (*n* = 57)	Low (*n* = 21)	NS (*n* = 12)	
Age				0.908
<60	29	11	7	
≥60	28	10	5	
Gender				0.778
Male	36	14	10	
Female	21	7	2	
Tumor differentiation				0.003**
Poor	24	2	1	
Moderate	22	10	8	
Well	11	9	3	
Tumor size (cm)				0.348
≤7	40	17	12	
>7	17	4	0	
Metastasis (LN)				0.391
No	55	21	12	
Yes	2	0	0	

*LN* Lymph node.

**p < 0.01.

**Table 2:** Univariate and multivariate analyses of factors associated with the overall survival in 78 ccRCC patients with significantly higher or lower expression of circPLIN2.

Factors	Overall survival					
	Univariate analysis			Multivariate analysis		
	HR	95% CI	*P* value	HR	95% CI	*P* value
Age	0.334	0.153–0.726	0.006**	0.306	0.129–0.725	0.007**
<60						
≥60						
Gender	0.943	0.458–1.944	0.875	1.414	0.634–3.157	0.397
Male						
Female						
Tumor differentiation	2.692	1.548–4.679	0.000***	1.882	1.021–3.469	0.043*
Poor						
Moderate						
Well						
Tumor size (cm)	0.431	0.211–0.882	0.021*	0.917	0.389–2.162	0.843
≤7						
>7						
Metastasis (LN)	0.089	0.019–0.433	0.003**	0.596	0.096–3.711	0.579
No						
Yes						
Tumor stage (AJCC)	2.298	1.515–3.487	0.000***	2.114	1.238–3.610	0.006**
1						
2						
3						
4						
circPLIN2 expression	0.304	0.106–0.869	0.026*	0.480	0.154–1.496	0.206
High						
Low						

Statistical analysis, Cox proportional hazard regression model; 95% CI, 95% confidence interval.

*HR* hazard ratio, *LN* lymph node.

*p < 0.05; **p < 0.01; and ***p < 0.001, which are considered significant differences.

### circPLIN2 promotes the proliferation, migration, and invasion of ccRCC cells in vitro

**Fig. 3:**
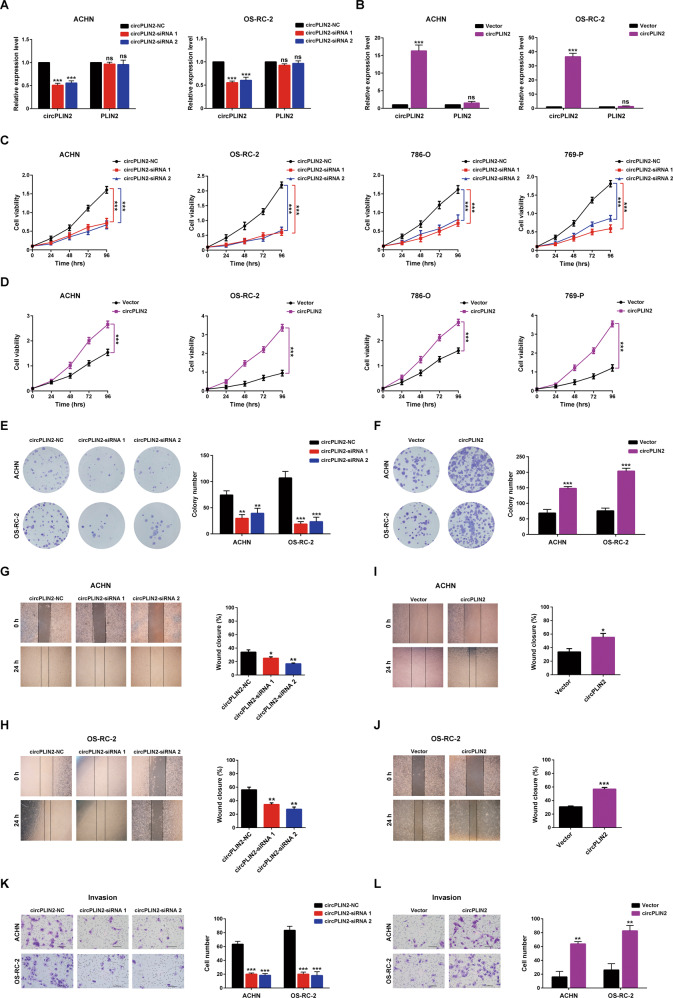
circPLIN2 promotes the proliferation, migration, and invasion of ccRCC cells in vitro. **A** RT–qPCR analysis of the relative expression levels of circPLIN2 and PLIN2 in ACHN and OS-RC-2 cells transfected with circPLIN2-siRNA 1/2 or circPLIN2-NC. **B** RT–qPCR analysis of the relative expression levels of circPLIN2 and PLIN2 in ACHN and OS-RC-2 cells transfected with circPLIN2 or vector. **C** CCK-8 cell viability assays for ACHN, OS-RC-2, 786-O and 769-P cells transfected with circPLIN2-siRNA 1/2 or circPLIN2-NC. **D** CCK-8 cell viability assays for ACHN, OS-RC-2, 786-O, and 769-P cells transfected with circPLIN2 or vector. **E** Colony formation assays for ACHN and OS-RC-2 cells transfected with circPLIN2-siRNA 1/2 or circPLIN2-NC. The number of colonies was determined (right panel). **F** Colony formation assays for ACHN and OS-RC-2 cells transfected with circPLIN2 or vector. The number of colonies was determined (right panel). **G**, **H** Wound-healing assays for ACHN (**G**) and OS-RC-2 (**H**) cells transfected with circPLIN2-siRNA 1/2 or circPLIN2-NC. The wound closure rate was calculated (right panel). Magnification, ×40. **I, J** Wound-healing assays for ACHN (**I**) and OS-RC-2 (**J**) cells transfected with circPLIN2 or vector. The wound closure rate was calculated (right panel). Magnification, ×40. **K** Matrigel-coated Transwell assays for ACHN and OS-RC-2 cells transfected with circPLIN2-siRNA 1/2 or circPLIN2-NC. The cell number per field was quantified (right panel). Scale bar, 100 μm. **L** Matrigel-coated Transwell assays for ACHN and OS-RC-2 cells transfected with circPLIN2 or vector. The cell number per field was quantified (right panel). Scale bar, 100 μm. Two-tailed Student’s t test. The error bars represent S.D. *p < 0.05, **p < 0.01, and ***p < 0.001.

To investigate whether changes in the expression of circPLIN2 affected the biological behaviors of ccRCC cells, two small interfering RNAs (circPLIN2-siRNA 1 and circPLIN2-siRNA 2) were designed and synthesized specifically targeting the back-splice junction of circPLIN2, and a circPLIN2 overexpression vector was designed and constructed. The results of RT–qPCR assays showed that these two siRNAs specifically knocked down circPLIN2 expression in ACHN and OS-RC-2 cells but had no effect on PLIN2 mRNA expression (Fig. 3A). Similarly, circPLIN2 was successfully overexpressed in ACHN and OS-RC-2 cells, while PLIN2 mRNA expression showed no obvious change (Fig. 3B). Then, we detected the effects of circPLIN2 knockdown and overexpression on the proliferation of ccRCC cells. The results of the CCK-8 assays showed that circPLIN2 knockdown significantly inhibited the proliferation of ACHN, OS-RC-2, 786-O, and 769-P cells (Fig. 3C), while circPLIN2 overexpression substantially promoted the proliferation of ACHN, OS-RC-2, 786-O and 769-P cells (Fig. 3D). Similar results were obtained in the colony formation assays. Knockdown of circPLIN2 markedly impaired the ability of ACHN and OS-RC-2 cells to form colonies (Fig. 3E), while overexpression of circPLIN2 notably enhanced the colony formation ability of ACHN and OS-RC-2 cells (Fig. 3F). Furthermore, wound-healing assays indicated that circPLIN2 knockdown significantly suppressed the migration of ACHN (Fig. 3G) and OS-RC-2 (Fig. 3H) cells, while circPLIN2 overexpression significantly enhanced the migration of ACHN (Fig. 3I) and OS-RC-2 (Fig. 3J) cells. In addition, Matrigel-coated Transwell assays showed that circPLIN2 knockdown obviously attenuated the invasion of ACHN and OS-RC-2 cells (Fig. 3K), and the opposite results were observed when circPLIN2 was overexpressed in ACHN and OS-RC-2 cells (Fig. 3L). Taken together, circPLIN2 significantly promoted the proliferation, migration, and invasion of ccRCC cells in vitro.

### circPLIN2 regulates the stability of the c-Myc and MARCKSL1 mRNAs by binding to the KH domains of IGF2BP proteins

**Fig. 4:**
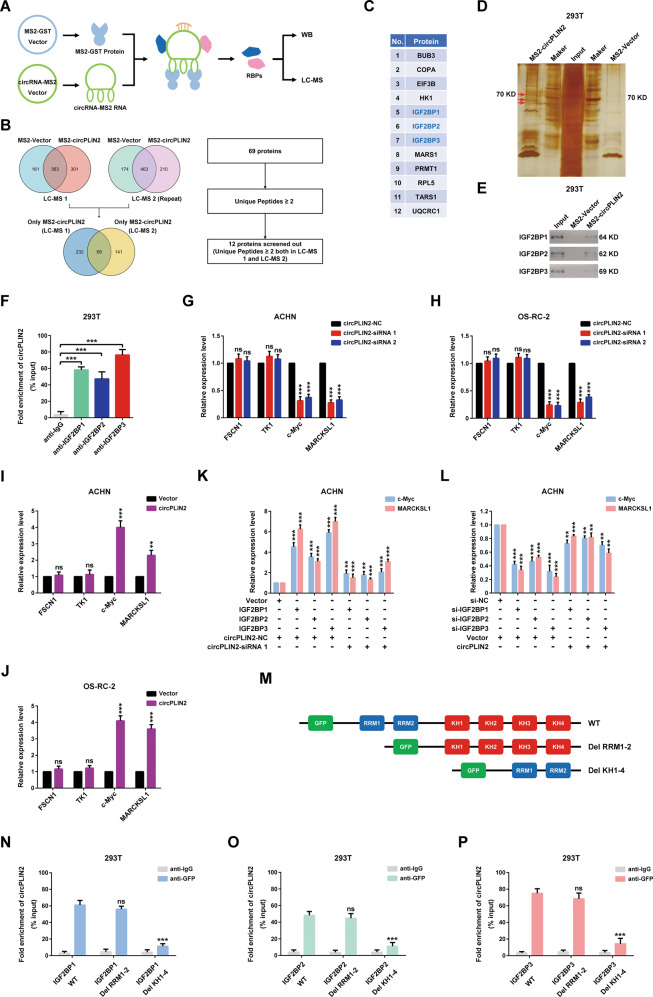
circPLIN2 regulates the stability of the c-Myc and MARCKSL1 mRNAs by binding to the KH domains of IGF2BP proteins. **A** Schematic diagram of tagged RNA affinity purification assays for the detection of proteins bound to circPLIN2 in 293T cells. **B** The flowchart delineates the steps of the LC–MS analysis for identifying proteins bound to circPLIN2 following tagged RNA affinity purification assays. LC–MS, liquid chromatography–mass spectrometry. **C** The chart for 12 proteins (unique peptides ≥ 2 both in LC–MS 1 and LC–MS 2) screened from LC–MS results. **D** Separation on SDS–PAGE gels and silver staining assays of the protein pulldown samples from 293T cells following tagged RNA affinity purification assays. **E** Western blot analysis of IGF2BP1, IGF2BP2, and IGF2BP3 protein levels in the protein pulldown samples from 293T cells following tagged RNA affinity purification assays. **F** RNA immunoprecipitation analysis of the fold enrichment of circPLIN2 with anti-IGF2BP1 antibody, anti-IGF2BP2 antibody or anti-IGF2BP3 antibody in 293T cells. The anti-IgG group was used as the control. **G**, **H** RT–qPCR analysis of the relative expression levels of FSCN1, TK1, c-Myc, and MARCKSL1 in ACHN (**G**) and OS-RC-2 (**H**) cells transfected with circPLIN2-siRNA 1/2 or circPLIN2-NC. **I, J** RT–qPCR analysis of the relative expression levels of FSCN1, TK1, c-Myc, and MARCKSL1 in ACHN (**I**) and OS-RC-2 (**J**) cells transfected with circPLIN2 or vector. **K** RT–qPCR analysis of the relative expression levels of c-Myc and MARCKSL1 in ACHN cells transfected with IGF2BPs or vector and circPLIN2-siRNA 1 or circPLIN2-NC. **L** RT–qPCR analysis of the relative expression levels of c-Myc and MARCKSL1 in ACHN cells transfected with si-IGF2BPs or si-NC and circPLIN2 or vector. **M** Schematic diagram of wild-type and truncated IGF2BP protein plasmids with GFP tags. WT, wild type. Del RRM1-2, deletion of the RRM1 and RRM2 domains in IGF2BP proteins. Del KH1-4, deletion of the KH1-4 domains in IGF2BP proteins. **N**–**P** RNA immunoprecipitation analysis of the fold enrichment of circPLIN2 with anti-GFP antibody or anti-IgG antibody in 293T cells transfected with IGF2BPs WT or IGF2BPs Del RRM1-2 or IGF2BPs Del KH1-4 vector. The anti-IgG group served as the control. IGF2BP1 (**N**), IGF2BP2 (**O**), and IGF2BP3 (**P**). Two-tailed Student’s t test. The error bars represent S.D. ns, not significant; **p < 0.01 and ***p < 0.001.

CircRNAs have been shown to interact with RNA-binding proteins to regulate protein functions [15–20]. As circPLIN2 was mainly distributed in the cytoplasm, RNA pull-down assays (tagged RNA affinity purification assays) were performed to identify the proteins that bound to circPLIN2 in the cytoplasm, and then purified proteins were subjected to liquid chromatography–mass spectrometry (LC–MS) and western blot analyses (Fig. 4A). To make the results of LC–MS more reliable, we repeated the LC–MS analysis and used a stricter screening standard (unique peptide≥2) to analyze the results of the two LC–MS experiments, and 12 proteins bound to circPLIN2 were screened (Fig. 4B, C and Supplementary Table 3). In addition, purified proteins obtained from RNA pull-down assays were subjected to separation on SDS–PAGE gels and subsequent silver staining, and the results were shown in Fig. 4D. Notably, there were three specific silver-stained bands at approximately 70 kD appearing in the MS2-circPLIN2 lane compared to the control MS2-Vector lane (Fig. 4D). Combined with the molecular weight and intracellular localization of the proteins (Supplementary Table 3), we speculated that these three specific silver-stained bands may be the IGF2BP1, IGF2BP2 and IGF2BP3 proteins. Accordingly, we performed western blot assays for the purified proteins obtained from RNA pull-down assays to detect the IGF2BP1, IGF2BP2 and IGF2BP3 proteins. The results indicated that circPLIN2 interacted with IGF2BP proteins (Fig. 4E and Supplementary Fig. 1A), consistent with the results of the RNA immunoprecipitation assays (Fig. 4F and Supplementary Fig. 2A, B).

Intriguingly, IGF2BP proteins can enhance the mRNA stability of downstream genes [35]. Therefore, we considered whether the interaction between circPLIN2 and IGF2BP proteins affected the mRNA stability of downstream genes of IGF2BP proteins, such as FSCN1, TK1, c-Myc, and MARCKSL1 [35]. RT–qPCR assays showed that circPLIN2 knockdown significantly reduced the c-Myc and MARCKSL1 mRNA levels but had no effect on the FSCN1 and TK1 mRNA levels (Fig. 4G, H). Similarly, circPLIN2 overexpression drastically increased the c-Myc and MARCKSL1 mRNA levels, while the FSCN1 and TK1 mRNA levels did not change significantly (Fig. 4I, J). Furthermore, rescue assays showed that circPLIN2 knockdown markedly reduced the c-Myc and MARCKSL1 mRNA levels that were increased by overexpressing IGF2BP proteins (Fig. 4K and Supplementary Fig. 3A), whereas circPLIN2 overexpression obviously increased the c-Myc and MARCKSL1 mRNA levels that were reduced by the knockdown of IGF2BP proteins (Fig. 4L and Supplementary Fig. 3B), suggesting that knockdown or overexpression of circPLIN2 apparently reversed the increases in the stability of the c-Myc and MARCKSL1 mRNAs induced by overexpression of IGF2BP proteins or decreases induced by knockdown of IGF2BP proteins. Intriguingly, neither knockdown nor overexpression of IGF2BP proteins altered the expression of circPLIN2 (Supplementary Fig. 4A–D), suggesting that the changes in IGF2BP protein expression may have no effect on the function of circPLIN2, as determined by changes in its expression. Based on these data, the binding of circPLIN2 to IGF2BP proteins increased the stability of the c-Myc and MARCKSL1 mRNAs.

We constructed GFP-tagged wild-type and truncated IGF2BP plasmids to further investigate the specific circPLIN2-binding domains of IGF2BP proteins (Fig. 4M). IGF2BP proteins have six key domains, including RRM1-2 domains and KH1-4 domains, and RNA immunoprecipitation assays showed that the enrichment of circPLIN2 was significantly reduced following the removal of the KH1-4 domains of the IGF2BP proteins, indicating that the KH1-4 domains were required for circPLIN2 to directly bind to the IGF2BP proteins (Fig. 4N–P). Interestingly, the KH domains were the key domains for the binding of IGF2BP proteins to downstream target genes [35], suggesting that the KH domains may provide a common site for binding among circPLIN2, IGF2BP proteins, and c-Myc or MARCKSL1 mRNA. Collectively, these data suggested that circPLIN2 increased the stability of the c-Myc and MARCKSL1 mRNAs by binding to the KH domains of IGF2BP proteins.

### Overexpression of c-Myc or MARCKSL1 alleviates the inhibitory effect of circPLIN2 knockdown on the proliferation, migration, and invasion of ccRCC cells in vitro

**Fig. 5:**
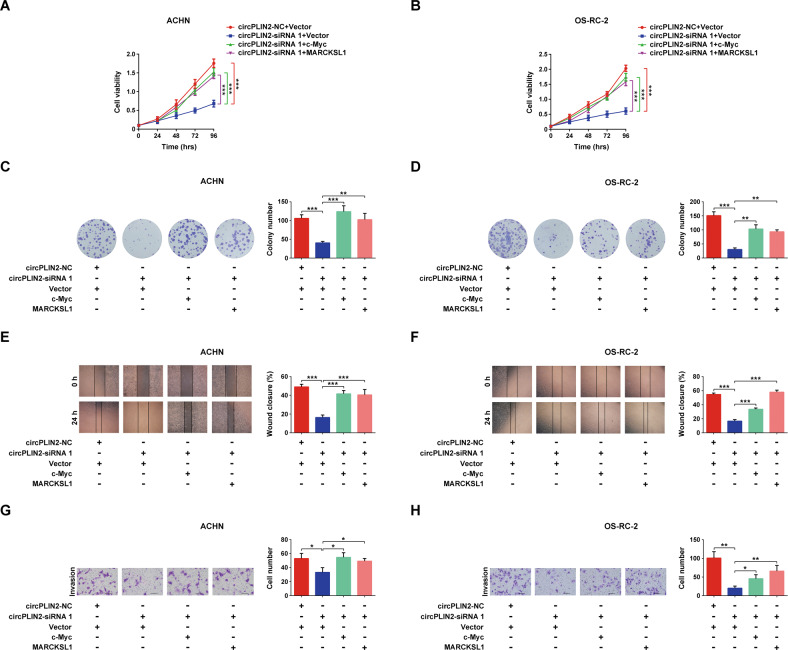
Overexpression of c-Myc or MARCKSL1 alleviates the inhibitory effects of circPLIN2 knockdown on the proliferation, migration, and invasion of ccRCC cells in vitro. **A**, **B** CCK-8 cell viability assays for ACHN (**A**) and OS-RC-2 (**B**) cells transfected with circPLIN2-siRNA 1 or circPLIN2-NC and c-Myc/MARCKSL1 or vector. **C**, **D** Colony formation assays for ACHN (**C**) and OS-RC-2 (**D**) cells transfected with circPLIN2-siRNA 1 or circPLIN2-NC and c-Myc/MARCKSL1 or vector. The number of colonies was determined. **E**, **F** Wound-healing assays for ACHN (**E**) and OS-RC-2 (**F**) cells transfected with circPLIN2-siRNA 1 or circPLIN2-NC and c-Myc/MARCKSL1 or vector. The wound closure rate was calculated. Magnification, ×40. **G**, **H** Matrigel-coated Transwell assays for ACHN (**G**) and OS-RC-2 (**H**) cells transfected with circPLIN2-siRNA 1 or circPLIN2-NC and c-Myc/MARCKSL1 or vector. The cell number per field was quantified. Scale bar, 100 μm. Two-tailed Student’s t test. The error bars represent S.D. **p < 0.01 and ***p < 0.001.

We constructed c-Myc and MARCKSL1 overexpression vectors to explore their involvement in the circPLIN2-regulated development and progression of ccRCC. CCK-8 cell viability assays showed that circPLIN2 knockdown significantly inhibited the proliferation of ACHN and OS-RC-2 cells, while overexpression of c-Myc or MARCKSL1 significantly promoted the proliferation of ACHN and OS-RC-2 cells, suggesting that overexpression of c-Myc or MARCKSL1 rescued the inhibitory effect of circPLIN2 knockdown on the proliferation of ccRCC cells (Fig. 5A, B). Similar results were obtained in the colony formation assays. Overexpression of c-Myc or MARCKSL1 rescued the long-term inhibitory effect of circPLIN2 knockdown on the proliferation of ccRCC cells (Fig. 5C, D). Furthermore, the results of wound-healing assays showed that circPLIN2 knockdown substantially decreased the wound-healing speed of ACHN and OS-RC-2 cells, while overexpression of c-Myc or MARCKSL1 markedly accelerated the wound-healing speed of ACHN and OS-RC-2 cells, revealing that overexpression of c-Myc or MARCKSL1 rescued the inhibitory effect of circPLIN2 knockdown on the migration of ccRCC cells (Fig. 5E, F). In addition, the Matrigel-coated Transwell assays indicated that overexpression of c-Myc or MARCKSL1 significantly rescued the suppressive effect of circPLIN2 knockdown on the invasion of ccRCC cells in vitro (Fig. 5G, H). Taken together, overexpression of c-Myc or MARCKSL1 rescued the inhibitory effect of circPLIN2 knockdown on the proliferation, migration, and invasion of ccRCC cells in vitro, suggesting that c-Myc and MARCKSL1 mediated the regulatory effects of circPLIN2 on ccRCC development and progression.

### circPLIN2 competitively sponges miR-199a-3p to abolish its repressive effect on ZEB1 expression

**Fig. 6:**
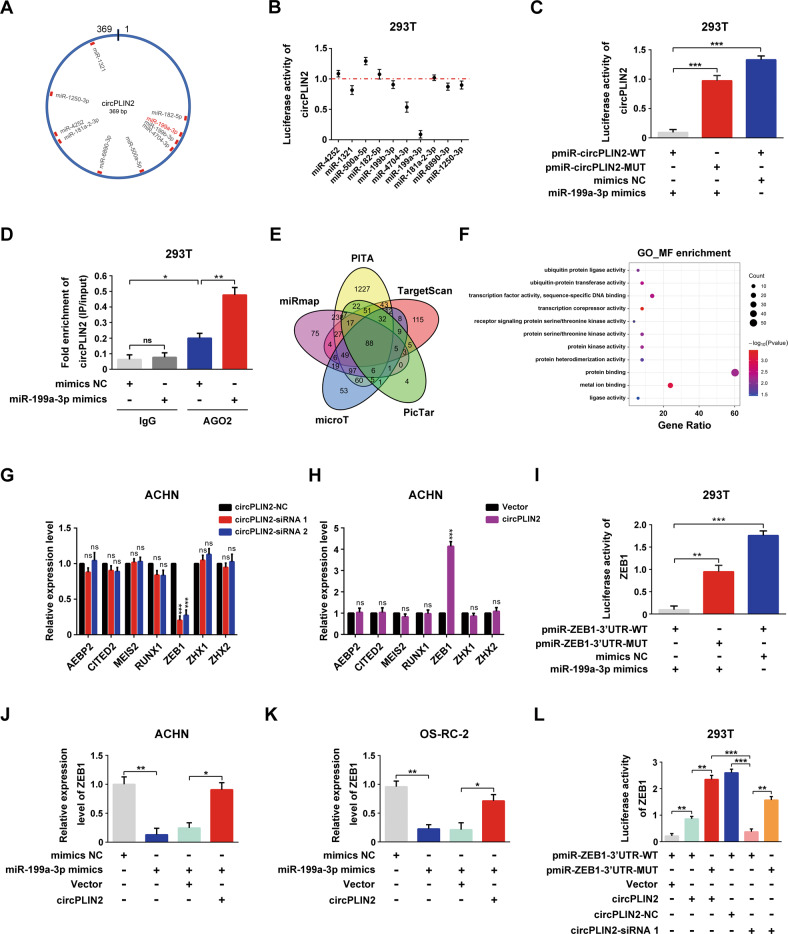
circPLIN2 competitively sponges miR-199a-3p to abolish the repressive effect of miR-199a-3p on ZEB1 expression. **A** A sketch map was drawn to show circPLIN2 sponging 10 miRNAs predicted in the circBank database. **B** Dual-luciferase reporter assays for the luciferase activity of circPLIN2 in 293T cells transfected with different miRNAs. Luciferase activity was normalized to firefly luciferase activity. **C** Dual-luciferase reporter assays for the luciferase activity of circPLIN2 in 293T cells transfected with pmiR-circPLIN2-WT or pmiR-circPLIN2-MUT and mimics NC or miR-199a-3p mimics. Luciferase activity was normalized to firefly luciferase activity. **D** RNA immunoprecipitation analysis of the fold enrichment of circPLIN2 with an anti-AGO2 antibody or anti-IgG antibody in 293T cells transfected with mimics NC or miR-199a-3p mimics. The IgG group served as the control. AGO2, Argonaute 2. **E** Venn diagram showing the downstream target genes of miR-199a-3p predicted by the TargetScan, PicTar, microT, miRmap, and PITA databases. **F** GO_MF enrichment analysis of 88 downstream target genes of miR-199a-3p commonly predicted in the TargetScan, PicTar, microT, miRmap, and PITA databases. GO, Gene Ontology. MF, Molecular function. **G** RT–qPCR analysis of the relative expression levels of AEBP2, CITED2, MEIS2, RUNX1, ZEB1, ZHX1, and ZHX2 in ACHN cells transfected with circPLIN2-siRNA 1/2 or circPLIN2-NC. **H** RT–qPCR analysis of the relative expression levels of AEBP2, CITED2, MEIS2, RUNX1, ZEB1, ZHX1, and ZHX2 in ACHN cells transfected with circPLIN2 or vector. **I** Dual-luciferase reporter assays for the luciferase activity of ZEB1 in 293T cells transfected with pmiR-ZEB1-3′UTR-WT or pmiR-ZEB1-3′UTR-MUT and mimics NC or miR-199a-3p mimics. Luciferase activity was normalized to firefly luciferase activity. **J**, **K** RT–qPCR analysis of the relative expression levels of ZEB1 in ACHN (**J**) and OS-RC-2 (**K**) cells transfected with mimics NC or miR-199a-3p mimics and circPLIN2 or vector. **L** Dual-luciferase reporter assays for the luciferase activity of ZEB1 in 293 T cells transfected with pmiR-ZEB1-3′UTR-WT or pmiR-ZEB1-3′UTR-MUT and circPLIN2 or vector, as well as circPLIN2-siRNA 1 or circPLIN2-NC. Luciferase activity was normalized to firefly luciferase activity. Two-tailed Student’s t test. The error bars represent S.D. ns, not significant; *p < 0.05, **p < 0.01, and ***p < 0.001.

Based on accumulating evidence, circRNAs function as sponges for miRNAs to regulate gene expression through the competing endogenous RNA (ceRNA) mechanism [11–14]. As circPLIN2 was preferentially distributed in the cytoplasm (Fig. 1H–K), we investigated whether circPLIN2 might also function through a ceRNA mechanism. We first made predictions using the circBank (http://www.circbank.cn/index.html) database and selected 10 miRNAs that might be sponged by circPLIN2 for further validation (Fig. 6A). The dual-luciferase reporter assays showed that miR-199a-3p exerted a particularly significant inhibitory effect on the luciferase activity of circPLIN2, suggesting that circPLIN2 might sponge miR-199a-3p (Fig. 6B). We constructed a circPLIN2 dual-luciferase reporter with the mutated miR-199a-3p binding site to further verify that circPLIN2 sponged miR-199a-3p (Supplementary Fig. 5A). The results of dual-luciferase reporter assays showed that wild-type (WT) circPLIN2 luciferase activity was significantly inhibited by miR-199a-3p, while mutated (MUT) circPLIN2 luciferase activity was not affected (Fig. 6C). In addition, the results of RNA immunoprecipitation assays showed that circPLIN2 was drastically enriched on AGO2 protein compared with the control IgG, and the enrichment of circPLIN2 on AGO2 protein was further increased when miR-199a-3p was added (Fig. 6D). These data revealed that circPLIN2 sponged miR-199a-3p (Fig. 6A–D).

Next, we predicted the target genes of miR-199a-3p using the TargetScan (https://www.targetscan.org/), PicTar (https://pictar.mdc-berlin.de/), microT (https://mrmicrot.imsi.athenarc.gr/), miRmap (https://mirmap.ezlab.org/), and PITA (https://genie.weizmann.ac.il/pubs/mir07/index.html) databases. We identified 88 target genes that coappeared in these five databases (Fig. 6E). We further performed an enrichment analysis of the Gene Ontology molecular functions (GO_MF enrichment) of these 88 target genes of miR-199a-3p using the DAVID tool (https://david.ncifcrf.gov/) and found that the *P* value of the “transcription corepressor activity” term was the most significant (*P* = 0.000351) (Fig. 6F). Seven target genes of miR-199a-3p appeared in the “transcription corepressor activity” term, including AEBP2, CITED2, MEIS2, RUNX1, ZEB1, ZHX1, and ZHX2. According to RT–qPCR assays, circPLIN2 knockdown substantially suppressed ZEB1 expression but had no effect on the expression levels of AEBP2, CITED2, MEIS2, RUNX1, ZHX1, and ZHX2 (Fig. 6G). Similarly, circPLIN2 overexpression significantly increased ZEB1 expression, while the expression levels of AEBP2, CITED2, MEIS2, RUNX1, ZHX1, and ZHX2 showed no obvious changes (Fig. 6H). Moreover, wild-type (WT) and mutated (MUT) ZEB1 dual-luciferase reporters targeting the miR-199a-3p binding site were constructed to detect the binding of ZEB1 and miR-199a-3p (Supplementary Fig. 5B). The results of the dual-luciferase reporter assays showed that the addition of miR-199a-3p significantly inhibited wild-type ZEB1 luciferase activity, while mutated ZEB1 luciferase activity was not affected, suggesting that ZEB1 sponged miR-199a-3p (Fig. 6I).

Next, we considered whether a ceRNA mechanism existed among circPLIN2, miR-199a-3p, and ZEB1. The RT–qPCR results showed that miR-199a-3p significantly reduced ZEB1 expression, while circPLIN2 overexpression abolished the repressive effect of miR-199a-3p on ZEB1 expression (Fig. 6J, K). In addition, the results of the dual-luciferase reporter assays indicated that circPLIN2 overexpression significantly increased wild-type ZEB1 luciferase activity, while circPLIN2 knockdown markedly decreased wild-type ZEB1 luciferase activity (Fig. 6L). Moreover, mutated ZEB1 luciferase activity was not affected by circPLIN2 overexpression or knockdown (Fig. 6L). These results revealed an endogenous RNA competition relationship between circPLIN2 and ZEB1 for miR-199a-3p. Collectively, these results suggested that circPLIN2 competitively sponged miR-199a-3p to abolish the repressive effect of miR-199a-3p on ZEB1 expression.

### circPLIN2 exerts its carcinogenic effects on ccRCC cells via the miR-199a-3p/ZEB1 axis in vitro

**Fig. 7:**
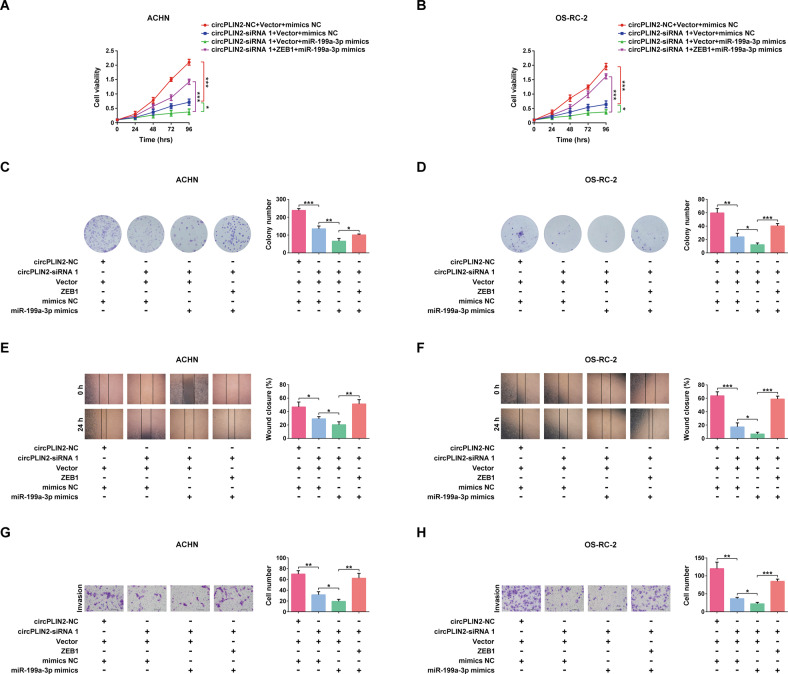
circPLIN2 exerts its carcinogenic effects on ccRCC cells via the miR-199a-3p/ZEB1 axis in vitro. **A**, **B** CCK-8 cell viability assays for ACHN (**A**) and OS-RC-2 (**B**) cells transfected with circPLIN2-siRNA 1 or circPLIN2-NC and ZEB1 or vector and miR-199a-3p mimics or mimics NC. **C**, **D** Colony formation assays for ACHN (**C**) and OS-RC-2 (**D**) cells transfected with circPLIN2-siRNA 1 or circPLIN2-NC and ZEB1 or vector and miR-199a-3p mimics or mimics NC. The number of colonies was determined. **E**, **F** Wound-healing assays for ACHN (**E**) and OS-RC-2 (**F**) cells transfected with circPLIN2-siRNA 1 or circPLIN2-NC and ZEB1 or vector and miR-199a-3p mimics or mimics NC. The wound closure rate was calculated. Magnification, ×40. **G**, **H** Matrigel-coated Transwell assays for ACHN (**G**) and OS-RC-2 (**H**) cells transfected with circPLIN2-siRNA 1 or circPLIN2-NC and ZEB1 or vector and miR-199a-3p mimics or mimics NC. The cell number per field was quantified. Scale bar, 100 μm. Two-tailed Student’s t test. The error bars represent S.D. *p < 0.05, **p < 0.01, and ***p < 0.001.

Next, we investigated whether the circPLIN2/miR-199a-3p/ZEB1 molecular signaling pathway participated in the development and progression of ccRCC. The results of CCK-8 cell viability assays showed that circPLIN2 knockdown significantly repressed the proliferation of ACHN and OS-RC-2 cells, and the proliferation of ACHN and OS-RC-2 cells was further inhibited when miR-199a-3p was added (Fig. 7A, B). Overexpression of ZEB1 rescued the inhibitory effects of circPLIN2 knockdown and the addition of miR-199a-3p on the proliferation of ccRCC cells (Fig. 7A, B). Similar results were obtained in the colony formation assays. ZEB1 overexpression drastically rescued the long-term suppressive effects of circPLIN2 knockdown and the addition of miR-199a-3p on the proliferation of ccRCC cells (Fig. 7C, D). Furthermore, the wound-healing assays indicated that circPLIN2 knockdown markedly reduced the wound-healing speeds of ACHN and OS-RC-2 cells, and the wound-healing speeds of ACHN and OS-RC-2 cells were even slower when miR-199a-3p was added, while ZEB1 overexpression significantly rescued the inhibitory effects of circPLIN2 knockdown and the addition of miR-199a-3p on the migration of ccRCC cells (Fig. 7E, F). Moreover, the results of Matrigel-coated Transwell assays indicated that ZEB1 overexpression noticeably rescued the repressive effects of circPLIN2 knockdown and the addition of miR-199a-3p on the invasion of ccRCC cells in vitro (Fig. 7G, H). Overall, our data suggested that the circPLIN2/miR-199a-3p/ZEB1 molecular signaling pathway was involved in the proliferation, migration, and invasion of ccRCC cells.

### circPLIN2 promotes ccRCC tumor growth and metastasis in vivo

**Fig. 8:**
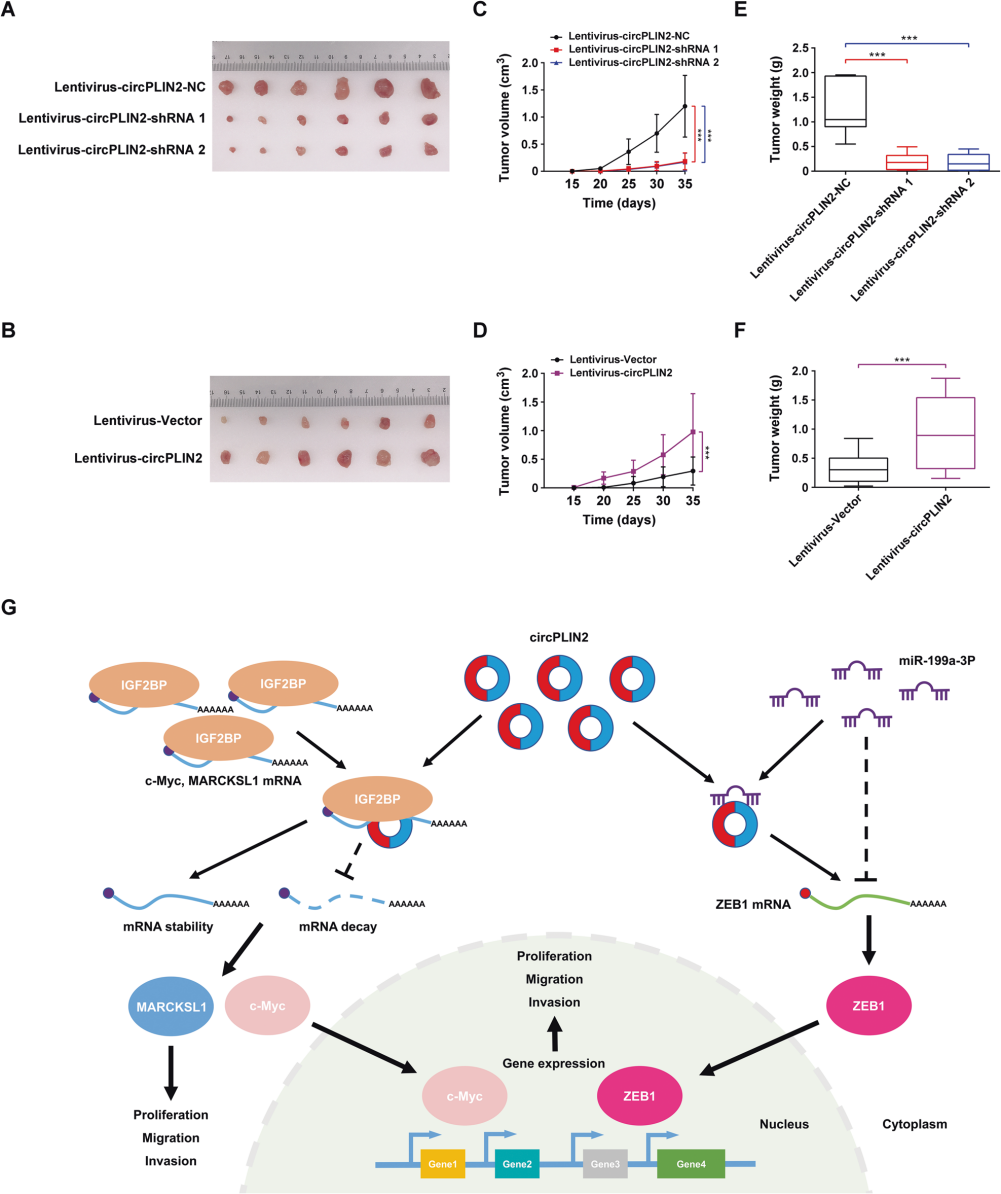
circPLIN2 promotes ccRCC tumor growth in vivo. **A** Following subcutaneous injections of ACHN cells transfected with lentivirus-circPLIN2-shRNA 1/2 or lentivirus-circPLIN2-NC in athymic nude mice and after monitoring tumor growth for 35 days, photographs of the tumors were obtained at necropsy. **B** Following subcutaneous injections of ACHN cells transfected with lentivirus-circPLIN2 or lentivirus-vector in athymic nude mice and after monitoring tumor growth for 35 days, photographs of the tumors were obtained at necropsy. **C**, **D** The volumes of subcutaneous xenograft tumors of ACHN cells were measured every 5 days. **E**, **F** Boxplot showing the weights of xenograft tumors established using ACHN cells that were isolated from nude mice 35 days after subcutaneous injection. **G** Hypothesis diagram illustrating the function and mechanism of circPLIN2 in ccRCC progression. Two-tailed Student’s t test. The error bars represent S.D. ***p < 0.001.

To examine the effect of circPLIN2 on the growth and metastasis of ccRCC cells in vivo, ACHN cells with stable low or high expression of circPLIN2 were injected into nude mice to establish a subcutaneous xenograft tumor model and a lung metastasis model. In the subcutaneous xenograft tumor model, stable knockdown of circPLIN2 significantly inhibited the growth of ACHN cells in vivo (Fig. 8A), while stable overexpression of circPLIN2 drastically promoted the growth of ACHN cells in vivo (Fig. 8B). In addition, the volumes of subcutaneous xenograft tumors indicated that stable knockdown of circPLIN2 markedly decreased the volumes of tumors in nude mice compared with the control group (Fig. 8C), whereas stable overexpression of circPLIN2 produced the opposite results (Fig. 8D), consistent with the results of weight measurement of subcutaneous xenograft tumors (Fig. 8E, F). In the lung metastasis model, suppression of circPLIN2 led to an apparent decrease in lung metastasis (Supplementary Fig. 6A, B), while circPLIN2 overexpression significantly promoted tumor metastasis in the lungs (Supplementary Fig. 6C, D). Furthermore, HE staining suggested fewer lung tumor foci and smaller volumes of lung metastatic nodules in the circPLIN2 knockdown group than in the control group (Supplementary Fig. 6E), whereas the circPLIN2 overexpression group displayed the opposite results (Supplementary Fig. 6F). Collectively, circPLIN2 may play an important role in promoting the growth and metastasis of ccRCC cells in vivo.

## Discussion

In this study, we indicated the oncogenic roles of circPLIN2 and determined its underlying mechanism in the development and progression of ccRCC. We first explored the circRNA expression profiles in 10 paired samples of RCC from GSE124453 and GSE108735 in the GEO database. We initially identified hsa_circ_0086457, designated circPLIN2, derived from exons 4 to 5 of the PLIN2 gene. We found that circPLIN2 was preferentially distributed in the cytoplasm of ccRCC cells and had a longer half-life and a stronger resistance to RNase R digestion than its linear counterpart PLIN2. The expression of circPLIN2 was significantly upregulated in ccRCC cells and tissues, and its overexpression was correlated with higher clinical stage and worse prognosis for ccRCC patients. Intriguingly, depletion of circPLIN2 significantly attenuated the proliferation, migration, and invasion of ccRCC cells in vitro and ccRCC tumor growth and metastasis in vivo, whereas overexpression of circPLIN2 produced the opposite effects, suggesting that elevated circPLIN2 expression may be a cancer-promoting event in ccRCC. Mechanistically, circPLIN2 not only increased the stability of the c-Myc and MARCKSL1 mRNAs by binding to the KH domains of IGF2BP proteins but also competitively sponged miR-199a-3p to abolish the repressive effect of miR-199a-3p on ZEB1 expression, ultimately resulting in ccRCC tumorigenesis and progression. Together, these findings indicated the oncogenic function of circPLIN2 and its potential molecular mechanism in which elevated circPLIN2 participated in the development and progression of ccRCC by binding IGF2BP proteins and miR-199a-3p to regulate the expression of their target genes, including c-Myc, MARCKSL1, and ZEB1 (Fig. 8G).

According to recent evidence, circRNAs play vital roles in the development and progression of ccRCC [36–39]. For example, circZNF609, which is highly expressed in various ccRCC cell lines, acts as a sponge for miR-138-5p to upregulate FOXP4 expression and promote the growth and invasion of ccRCC [36]. Intriguingly, circZNF609 can be translated into a functional small protein in myoblasts [40], which is regulated by its own m^6^A modification [41]. Hence, we consider whether circZNF609 is translated into a protein and plays regulatory role in ccRCC, which requires further exploration in the future. As another example showed, circTLK1 was not merely substantially upregulated in ccRCC cells and tissues but was related to the distant metastasis of tumors and the prognosis of ccRCC patients [37]. Moreover, circTLK1 upregulated CBX4 expression by competitively sponging miR-136-5p to exert its oncogenic activity [37]. Although these circRNAs have been shown to be involved in the development and progression of ccRCC, their key regulatory roles and molecular mechanisms have not been fully clarified. In addition, novel circRNAs must be further identified in ccRCC.

In this study, we selected 10 paired RCC samples with circRNA expression data from GSE124453 and GSE108735 in the GEO database for joint analysis to more accurately detect the expression profiles of circRNAs in RCC, which reduced the bias associated with RCC sample selection in two different studies and expanded the RCC sample size to ensure that circRNA expression data more reliable. We found that circPLIN2, an oncogene, was significantly highly expressed in ccRCC cells and tissues, and its overexpression was correlated with higher clinical stage and worse prognosis for ccRCC patients. Furthermore, circPLIN2 promoted ccRCC cell proliferation, migration, and invasion in vitro and ccRCC tumor growth and metastasis in vivo. These results are similar to the performance and function of circTLK1, circSDHC, and circPRRC2A in ccRCC [37, 42, 43]. However, unlike circPLIN2, circRAPGEF5 and circAKT3 were expressed at significantly lower levels in ccRCC and inhibited the malignant progression of ccRCC [44, 45]. Overall, these conflicting results for circRNA performance in ccRCC may be partially explained by the fact that circRNAs participate in different molecular signaling pathways.

Notably, based on the results predicted using the circBank database, although circPLIN2 contains an open reading frame (ORF), circPLIN2 lacks the internal ribosome entry site (IRES) elements [46] and m^6^A (N^6^-methyladenosine) modification [24, 47] required for the translation of circRNAs [48]; therefore, circPLIN2 may not have translation potential. We speculate that circPLIN2 may regulate the expression of downstream genes by binding to proteins or sponging miRNAs in the cytoplasm. In the present study, we described the binding of circPLIN2 and IGF2BP proteins, including IGF2BP1, IGF2BP2, and IGF2BP3 proteins. IGF2BP proteins, which are mainly enriched in the cytoplasm, recognize and bind target mRNAs in an m^6^A-dependent manner and function as stabilizers to inhibit the degradation of their target mRNAs [35]. The IGF2BP proteins contain six key domains, including the RRM1-2 domains and KH1-4 domains, and the KH1-4 domains are required for the binding of IGF2BP proteins and target mRNAs [35]. Interestingly, the specific circPLIN2-binding domains in IGF2BP proteins happened to be the KH1-4 domains, suggesting that the KH1-4 domains might represent a common site of action among circPLIN2, IGF2BP proteins, and the c-Myc or MARCKSL1 mRNA. In our study, circPLIN2 increased the stability of the c-Myc and MARCKSL1 mRNAs by binding to the KH domains of IGF2BP proteins. Moreover, the oncogenes c-Myc [49, 50] and MARCKSL1 [51, 52] participate in the development and progression of cancers. Subsequent rescue assays showed that overexpression of c-Myc or MARCKSL1 significantly rescued the inhibitory effects of circPLIN2 knockdown on the proliferation, migration, and invasion of ccRCC cells, suggesting that c-Myc and MARCKSL1 were involved in the circPLIN2-regulated development and progression of ccRCC.

In addition, we identified an underlying ceRNA mechanism in which circPLIN2 competitively sponged miR-199a-3p to abolish the repressive effect of miR-199a-3p on ZEB1 expression. Subsequent rescue assays further showed that the circPLIN2/miR-199a-3p/ZEB1 molecular signaling pathway participated in the development and progression of ccRCC. Intriguingly, ZEB1, a transcriptional repressor, inhibits E-cadherin transcription by recruiting BRG1 and promotes the epithelial–mesenchymal transition (EMT) and tumor progression [53]. Hence, we speculate that the EMT may be involved in the circPLIN2-regulated development and progression of ccRCC, which requires further evaluation. Moreover, ZEB1 suppresses the expression of stemness-inhibiting miR-200 and miR-203 and promotes tumor proliferation and progression [54]. Therefore, we speculate that miR-200 and miR-203 may also participate in circPLIN2-mediated ccRCC progression, which also needs to be further confirmed.

Notably, circRNAs play important roles in the multilevel regulation of gene expression, including by functioning as miRNA sponges [11–14], participating in RNA‒protein interactions [15–20], and through their protein-coding ability [21–26]. Intriguingly, in the present study, circPLIN2 bound to the KH domains of IGF2BP proteins to increase the stability of the c-Myc and MARCKSL mRNAs and promote the development and progression of ccRCC. Moreover, circPLIN2 also competitively sponged miR-199a-3p to abolish the repressive effect of miR-199a-3p on ZEB1 expression as a method to exert its carcinogenic effects on ccRCC. In fact, we prove that circPLIN2 binding to IGF2BP proteins and sponging of miR-199a-3p both play important roles in the development and progression of ccRCC. However, to date, we have not determined whether circPLIN2 binding to IGF2BP proteins or sponging of miR-199a-3p is more important for the development and progression of ccRCC, which requires further investigation in the future.

In conclusion, our study suggests that circPLIN2 functions as an oncogene and participates in the development and progression of ccRCC. In addition, circPLIN2 not only regulates the stability of the c-Myc and MARCKSL1 mRNAs by binding to the KH domains of IGF2BP proteins but also sponges miR-199a-3p to abolish the repressive effect of miR-199a-3p on ZEB1 expression, ultimately resulting in ccRCC tumorigenesis and progression. According to these data, circPLIN2 may serve as a promising diagnostic and prognostic biomarker and a potential therapeutic target for ccRCC patients.

## Materials and methods

### Bioinformatics analysis of the circRNA expression profile in RCC

We first retrieved circRNA expression data in RCC from the GEO database (http://www.ncbi.nlm.nih.gov/geo) and obtained the GSE124453 and GSE108735 datasets. Then, we downloaded the raw data from the GSE124453 and GSE108735 datasets from the SRA database (https://www.ncbi.nlm.nih.gov/sra) and converted them into FASTQ format using Sratoolkit software (version 2.9.2) (https://hpc.nih.gov/apps/sratoolkit.html). The FASTQ files were aligned to the human hg38 reference using STAR software (version 2.7.1a) (https://github.com/alexdobin/STAR) [55]. circRNAs were subsequently calculated and identified using DCC software (https://github.com/dieterich-lab/DCC) with the default parameters [56]. Next, the circRNAs identified were filtered by read count more than 5 and expressed samples over 30%. The function and identities of circRNAs were then annotated using the circBase database (http://www.circbase.org) [57]. DESeq2 was used to read the raw count matrix after filtration, and normalization was performed using the variance stabilizing transformation algorithm [58]. Significantly differentially expressed circRNAs between RCC and normal samples were screened with the criteria of adjusted p value less than 0.05 and absolute value of log_2_(fold change) more than 2. The results of the bioinformatics analysis were eventually visualized as a heatmap and a volcano plot.

### Plasmid construction and cell transfection assay

Referring to the method for constructing the circTP63 overexpression vector described in a previous study [59], we successfully constructed the circPLIN2 overexpression vector through homologous recombination using the pLCDH-ciR plasmid. For cell transfection assays, briefly, cells were first seeded on 6-well plates and grown to a confluence of ~50%. Next, cells were transfected with circPLIN2 or the vector using Lipofectamine 2000 reagent according to the manufacturer’s protocol and then cultured at 37 °C with 5% CO_2_ for 48–72 h. Finally, circPLIN2 expression was assessed using RT–qPCR. In addition, the c-Myc, MARCKSL1, and ZEB1 overexpression vectors were designed and constructed by GENE (Shanghai, China) using the GV658 plasmid. GFP-tagged wild-type and truncated IGF2BP vectors were designed and constructed by GENE using the pEGFP-C2 plasmid. The primers used for plasmid construction are listed in Supplementary Table 4.

### Xenograft model

For tumor formation assays in vivo, thirty female athymic BALB/c nude mice, aged 6–8 weeks and purchased from Gempharmatech (Nanjing, China), were raised in an SPF environment and received care according to the protocols. These nude mice were randomly and equally divided into lentivirus-circPLIN2-NC, lentivirus-circPLIN2-shRNA 1, lentivirus-circPLIN2-shRNA 2, lentivirus-vector and lentivirus-circPLIN2 groups. Next, 100 µl of ACHN cell suspension containing 1 × 10^6^ cells with stable high or low expression of circPLIN2 was subcutaneously injected into the right flank of each mouse in the corresponding group. The volumes of subcutaneous xenograft tumors were measured every 5 days beginning on the 15th day after injection. After 35 days, the nude mice were sacrificed by cervical dislocation, and the tumor tissues were measured, weighed, and photographed. In addition, the tumor volume was calculated as (length × width × height)/2. For tumor metastasis assays in vivo, twenty-four female athymic BALB/c nude mice aged 6–8 weeks were randomly and equally divided into lentivirus-circPLIN2-NC, lentivirus-circPLIN2-shRNA 1, lentivirus-vector and lentivirus-circPLIN2 groups. Briefly, 100 µl of ACHN cell suspension containing 1 × 10^6^ cells with stable high or low expression of circPLIN2 was injected into the tail vein of each mouse in the corresponding group. After 4 weeks of feeding, the nude mice were sacrificed, and the lung metastatic nodules were evaluated by a pathologist. Finally, the lung tissues were removed for hematoxylin-eosin (HE) staining. All animal assays were performed in accordance with animal use protocols approved by the Committee for the Ethics of Animal Experiments, Shenzhen Peking University - The Hong Kong University of Science and Technology Medical Center (protocol number 2021-565).

### Statistical analysis

The IBM SPSS package (version 23.0) and GraphPad Prism software (version 6.0) were used for statistical analyses. All data in this study are presented as the means ± S.D. of the values from triplicate assays. Two-tailed Student’s t test was used to compare two independent groups. Spearman’s test was performed to analyze the correlations for categorical variables. The Kaplan–Meier test was performed for the univariate analysis of overall survival, and the Cox proportional hazards regression model was used for the multivariate analysis of overall survival. *p < 0.05, **p < 0.01, and ***p < 0.001 were considered statistically significant.

### Reporting summary

Further information on research design is available in the Nature Research Reporting Summary linked to this article.

## Supplementary information

Supplementary Table 1

Supplementary Table 2

Supplementary Table 3

Supplementary Table 4

Supplementary Materials and methods

Supplementary Figure 1

Supplementary Figure 2

Supplementary Figure 3

Supplementary Figure 4

Supplementary Figure 5

Supplementary Figure 6

Supplementary Figure Legend

Certificate of English Language Editing

The completed reporting summary form

Supplementary LC-MS Results_MS2_Vector_1

Supplementary LC-MS Results_MS2_circPLIN2_1

Supplementary LC-MS Results_MS2_Vector_2

Supplementary LC-MS Results_MS2_circPLIN2_2

Supplementary LC-MS Results_Venn diagram_1

Supplementary LC-MS Results_Venn diagram_2

Supplementary LC-MS Results_Venn diagram_3_intersection

## Data Availability

The data underlying this article will be shared on reasonable request to the corresponding author.

## Code availability

The computer code used during the current study is available from the corresponding authors on reasonable request.
