# Supplementary material for: circPLIN2 promotes clear cell renal cell carcinoma progression by binding IGF2BP proteins and miR-199a-3p
Source: Cell Death Dis. 2022 Dec 9;13(12):1030. doi: 10.1038/s41419-022-05488-z (PMC9734136; doi:10.1038/s41419-022-05488-z)
Supplement: Supplementary file 5 — Supplementary Materials and methods [file 41419_2022_5488_MOESM5_ESM.docx]

**Materials and methods**

**Cells and cell culture**

Human ccRCC cells (ACHN and 786-O) were obtained from the Division of Life Sciences, School of Science, Hong Kong University of Science and Technology (Hong Kong, China). Human ccRCC cells (OS-RC-2 and 769-P) were purchased from Procell (Wuhan, China). Human immortalized proximal tubule epithelial cells (HK-2) and human embryonic kidney cells (293T) were obtained from the Biomedical Research Institute, Shenzhen Peking University - The Hong Kong University of Science and Technology Medical Center (Shenzhen, China). ACHN cells were cultured in MEM (Gibco, USA) supplemented with 10% fetal bovine serum (FBS) (Gibco, South America) at 37 °C with 5% CO_2_. OS-RC-2 and 769-P cells were cultured in RPMI-1640 (Gibco, USA) supplemented with 10% FBS at 37 °C with 5% CO_2_. HK-2 and 293T cells were cultured in DMEM (Gibco, USA) supplemented with 10% FBS at 37 °C with 5% CO_2_. All cells cultured from the fifth passage to the fifteenth passage were used in this study. All of the cell lines used in this study were ultimately obtained from American Type Culture Collection (ATCC) and authenticated by STR profiling and were free of mycoplasma contamination.

**Antibodies**

Antibodies against IGF2BP1 (8482S), IGF2BP2 (14672S), GFP (2956T) and AGO2 (2897T) were purchased from Cell Signaling Technology (Danvers, MA, USA). An antibody against IGF2BP3 (ab177477) was purchased from Abcam (Cambridge, UK). An antibody against IgG (GB111738) was purchased from Servicebio (Wuhan, China).

**PCR and agarose gel electrophoresis assays**

Total RNA was extracted from cells collected using RNA-easy isolation reagent (Vazyme, China) according to the manufacturer’s protocol. Then, cDNAs were synthesized using the GoScript Reverse Transcription System kit (Promega, USA) according to the manufacturer’s instructions. Moreover, gDNA was extracted from cells collected using a Genomic DNA Isolation Kit (TIANGEN, China) according to the manufacturer’s protocol. The cDNA and gDNA templates were subsequently used for PCR performed with a C1000 Thermal Cycler (Bio-Rad, USA) using primers specific for circPLIN2, PLIN2 and GAPDH (Supplementary Table 4). The cDNA and gDNA PCR products were finally observed after electrophoresis on 2% agarose gels.

**RT–qPCR assay**

Total RNA was extracted from the collected cells, and then cDNAs were synthesized. RT–qPCR was performed with a CFX96 Real-Time System (Bio-Rad, USA) using the iTaq Universal SYBR Green Supermix Kit (Bio-Rad, USA) according to the manufacturer’s protocol. The relative RNA expression levels were determined using the 2^−ΔCt^ or 2^−ΔΔCt^ method. GAPDH was used as a general control. The RT–qPCR primers are listed in Supplementary Table 4.

**RNase R digestion assay**

A total of 2.5 μg of total RNA extracted from 786-O cells was incubated at 37 °C for 30 min with or without RNase R (Geneseed, China) according to the manufacturer’s protocol and then analyzed using RT–qPCR following the inactivation of the RNase R enzyme at 70 °C for 10 min.

**Actinomycin D assay**

786-O cells were exposed to 2 μg/mL actinomycin D (Sigma, USA) at the indicated time points. Then, 786-O cells were collected, and total RNA was extracted. The stability of circPLIN2 and the PLIN2 mRNA was analyzed using RT–qPCR.

**Nuclear and cytoplasmic localization assay**

The nuclear and cytoplasmic fractions were extracted from cells using a PARIS kit (Ambion, USA) according to the manufacturer’s instructions. Subsequently, nuclear and cytoplasmic RNAs were converted to cDNAs, and then the abundance of circPLIN2 in the nuclear and cytoplasmic fractions was analyzed using RT–qPCR.

**Fluorescence in situ hybridization assay**

ACHN and OS-RC-2 cells were first seeded on 24-well plates and cultured to a confluence of approximately 50%. Then, fluorescence in situ hybridization (FISH) assays of ACHN and OS-RC-2 cells were performed using a FISH kit (Ribo, China) according to the manufacturer’s protocol. Briefly, cells were fixed with 4% paraformaldehyde for 15 min at room temperature and permeabilized with 1% Triton X-100 at 4 °C for 10 min. Next, the cells were blocked with the prehybridization solution at 37 °C for 30 min and then incubated with the hybridization solution containing the circPLIN2 probe mix (Supplementary Table 4) overnight at 37 °C in the dark. Subsequently, the cells were washed with SSC buffer and then incubated with DAPI in the dark for 5 min at room temperature. FISH images were acquired with a confocal microscope (LSM 710, ZEISS). In this study, Ribo designed and synthesized all FISH probe mixes and maintained the rights to the patent and final interpretation.

**In situ hybridization assay**

A tissue microarray (HKidE180Su03) of human ccRCC with follow-up data, including 90 cases of tumor tissues and adjacent tissues, was purchased from Outdo (Shanghai, China). In situ hybridization (ISH) staining was performed on the tissue microarray of ccRCC with probes specific for circPLIN2 to validate its expression. The circPLIN2 probes included primary and secondary probes (Supplementary Table 4). Briefly, the ccRCC tissue microarray was hybridized with the primary probe and then incubated with the secondary probe following dewaxing, hydration, proteinase K digestion and blockade of endogenous peroxidase activity. Subsequently, a mouse anti-digoxigenin-labeled horseradish peroxidase (anti-DIG-HRP) antibody and DAB reagent were added to the ccRCC tissue microarray. Images were successfully acquired after the ccRCC tissue microarray was counterstained with hematoxylin. ISH-stained tissues were scored by multiplying the extent (0-100) and intensity (0-3) of staining.

**RNA interference**

Ribo designed and synthesized two siRNAs specific for circPLIN2 (circPLIN2-siRNA 1 and circPLIN2-siRNA 2) and a negative control (circPLIN2-NC) and maintained the rights to the patent and final interpretation. Briefly, cells were cultured in 6-well plates to a confluence of approximately 50% and then transfected with circPLIN2-siRNA 1/2 or circPLIN2-NC using RNAiMAX reagent according to the manufacturer’s instructions. The transfected cells were cultured at 37 °C with 5% CO_2_ for 48 h, and the circPLIN2 expression level was subsequently detected using RT–qPCR. The siRNAs are listed in Supplementary Table 4.

**CCK-8 cell viability assay**

Cells were seeded on 96-well plates at a density of 2 x 10^3^ cells per well and then transfected for 6-8 hours after cell adherence. Next, CCK-8 cell viability assays were performed each day for the following 4 days. Briefly, 10 µl of CCK-8 reagent (MCE, USA) and 100 µl of complete medium were mixed and then added to each well. Subsequently, the cells were incubated at 37 °C for 2 h in the dark, and then the absorbance of the cell samples in each well was measured at 450 nm with a microplate reader.

**Colony formation assay**

Cells were seeded on 6-well plates at a density of 1 x 10^3^ cells per well and then transfected for 6-8 hours after cell adherence. Next, the cells were cultured in fresh complete medium for 12 days until visible cell colonies were formed. Subsequently, the cells were fixed with 75% ethanol for 10 min and then stained with 0.1% crystal violet for 15 min. The number of foci containing more than 30 cells was calculated.

**Wound-healing assay**

Cells were seeded evenly on 6-well plates and then transfected for 6-8 hours after cell adherence. After the transfected cells were cultured in fresh complete medium and grown to a confluence of approximately 80%, the cells were starved overnight. Next, a sterile 1000 µl plastic pipette tip was used to scratch the cells to create a linear wound, and then the cells were gently washed twice with PBS buffer to remove all cellular debris. Subsequently, 2 ml of fresh complete medium were added to each well, and then the cell wounds were photographed at 0 h under a microscope (CKX41, OLYMPUS). After culturing the cells for 24 h, photographs of the cell wounds were captured again under the same microscope. Finally, the healing rate of cell scratch wounds was calculated.

**Matrigel-coated Transwell assay**

The membranes of the upper chambers of Transwells in a 24-well plate (Corning, USA) were precoated with 50 µl of Matrigel (Corning, USA) diluted with serum-free medium overnight at 4 °C. Then, 200 µl of serum-free cell suspension containing 4 x 10^4^ transfected cells were added to the upper chamber of Transwells in a 24-well plate. The bottom chamber was filled with 500 µl of fresh complete medium. After culturing the cells at 37 °C for 24 h, the upper chamber was fixed with 100% methanol for 10 min and then stained with 0.1% crystal violet for 10 min. Cell invasion was photographed in three randomly selected fields under a microscope (80I, Nikon), and the number of cells penetrating the Matrigel and membrane was calculated.

**Tagged RNA affinity purification assay**

The purpose of tagged RNA affinity purification assays is to detect proteins or RNAs that bind to circRNAs. The MS2-circPLIN2 vector expressed circPLIN2 and the stem–loop structure of MS2 RNA and was constructed by Bersinbio (Guangzhou, China). Moreover, the MS2-GST vector expressed the MS2-GST fusion protein and was also constructed by Bersinbio. The MS2 vector or MS2-circPLIN2 vector was co-transfected with the MS2-GST vector into 293T cells to obtain the circPLIN2-MS2-GST complex based on the affinity of the MS2 RNA and MS2 protein. Subsequently, the complex was pulled down by glutathione magnetic beads. Finally, the proteins binding to circPLIN2 were eluted and purified, identified by liquid chromatography–mass spectrometry and verified by western blot.

**Liquid chromatography–mass spectrometry**

Thirty microliters of purified protein sample were analyzed by liquid chromatography–mass spectrometry (LC–MS) (GENE, China) following the tagged RNA affinity purification assays. We repeated the LC–MS detection. The results of the two LC–MS detections were analyzed, and a more stringent screening condition (unique peptide≥2) was adopted. The results of the LC–MS detection and analysis are provided in the supplementary LC–MS file.

**SDS–PAGE separation and silver staining**

After the tagged RNA affinity purification assays were performed, 15 µl of purified protein sample were separated on a 10% SDS–PAGE gel. Then, the SDS–PAGE gel was stained using a protein staining kit (Sangon, China) according to the manufacturer’s protocol and subsequently photographed.

**Western blot assay**

After the tagged RNA affinity purification assays were performed, 15 µl of purified protein sample were separated on a 10% SDS–PAGE gel and then transferred onto a PVDF membrane. Subsequently, the membrane was blocked with 5% nonfat powdered milk in TBST for 1 h at room temperature and then incubated with the indicated primary antibodies overnight at 4 °C, followed by an incubation with the secondary antibodies. Finally, signals were examined using the ultrasensitive ECL chemiluminescence kit (Servicebio, China) and the iBright FL1000 System (Invitrogen, USA).

**RNA immunoprecipitation assay**

RNA immunoprecipitation (RIP) assays were performed using a RIP kit (Geneseed, China) according to the manufacturer’s protocol. Briefly, cells were collected, washed twice with PBS buffer and then lysed. Next, magnetic beads were incubated with the indicated antibodies at 4 °C for 2 h. Then, the supernatant of the cell lysate was reacted with the antibody-bound magnetic beads overnight at 4 °C. Finally, circPLIN2 bound to the magnetic bead complex was eluted, purified and then analyzed using RT–qPCR.

**Dual-luciferase reporter assay**

For dual-luciferase assays, 293T cells were evenly plated in a 48-well plate. Then, the indicated dual-luciferase reporter plasmids and miRNA mimics were co-transfected into 293T cells following cell adherence. After transfected 293T cells were cultured for 24 h, dual-luciferase reporter assays were performed using a dual-luciferase reporter assay system kit (Promega, USA) according to the manufacturer’s instructions. Briefly, 293T cells were gently washed twice with PBS buffer and then lysed with 200 µl of 1×PLB for 15 min at room temperature. After centrifugation to remove cell debris, 10 µl of cell lysate were added to 40 µl of LAR II, and then firefly luciferase activity was measured with a microplate reader. Next, 40 µl of Stop&Glo were added to the cell lysate containing LAR II, and Renilla luciferase activity was measured with the same microplate reader. Finally, the ratio of Renilla luciferase activity to firefly luciferase activity in each sample was calculated. In this study, all reporter plasmids (pmiR-circPLIN2-WT, pmiR-circPLIN2-MUT, pmiR-ZEB1-3’UTR-WT and pmiR-ZEB1-3’UTR-MUT) were designed and synthesized by Ribo (Guangzhou, China). The Renilla luciferase gene in all reporter plasmids served as the reporter gene, while the firefly luciferase gene served as the reference gene. All miRNA mimics and mimics NC were synthesized by Ribo, which maintained the rights to the patent and final interpretation, and are listed in Supplementary Table 4.

**Generation of stable cell lines**

The circPLIN2 overexpression vector was constructed using the pLCDH-ciR plasmid and was then co-transfected with the packaging plasmid psPAX2 and the envelope plasmid pMD2.G into 293T cells. After 6-8 h, the cell culture medium was replaced with fresh complete medium, and then the 293T cells were incubated at 37 °C for 48 h. Next, the 293T cell supernatant was collected and centrifuged to remove impurities to obtain a lentivirus suspension. Subsequently, the quality of the collected lentiviral suspension, including its physical state, sterile state and titer, was checked. Finally, a qualified lentivirus suspension was obtained. Moreover, circPLIN2 RNAi lentiviral suspensions (lentivirus-circPLIN2-shRNA 1, lentivirus-circPLIN2-shRNA 2 and lentivirus-circPLIN2-NC) were obtained from GENE (Shanghai, China). For stable expression, briefly, ACHN cells were transfected with the lentivirus suspension and then treated with 2 µg/mL puromycin to generate cells with stable overexpression or knockdown of circPLIN2. Lentiviral target sequences are listed in Supplementary Table 4.
