# Supplementary figures and images for: circPLIN2 promotes clear cell renal cell carcinoma progression by binding IGF2BP proteins and miR-199a-3p
Source: Cell Death Dis. 2022 Dec 9;13(12):1030. doi: 10.1038/s41419-022-05488-z (PMC9734136; doi:10.1038/s41419-022-05488-z)

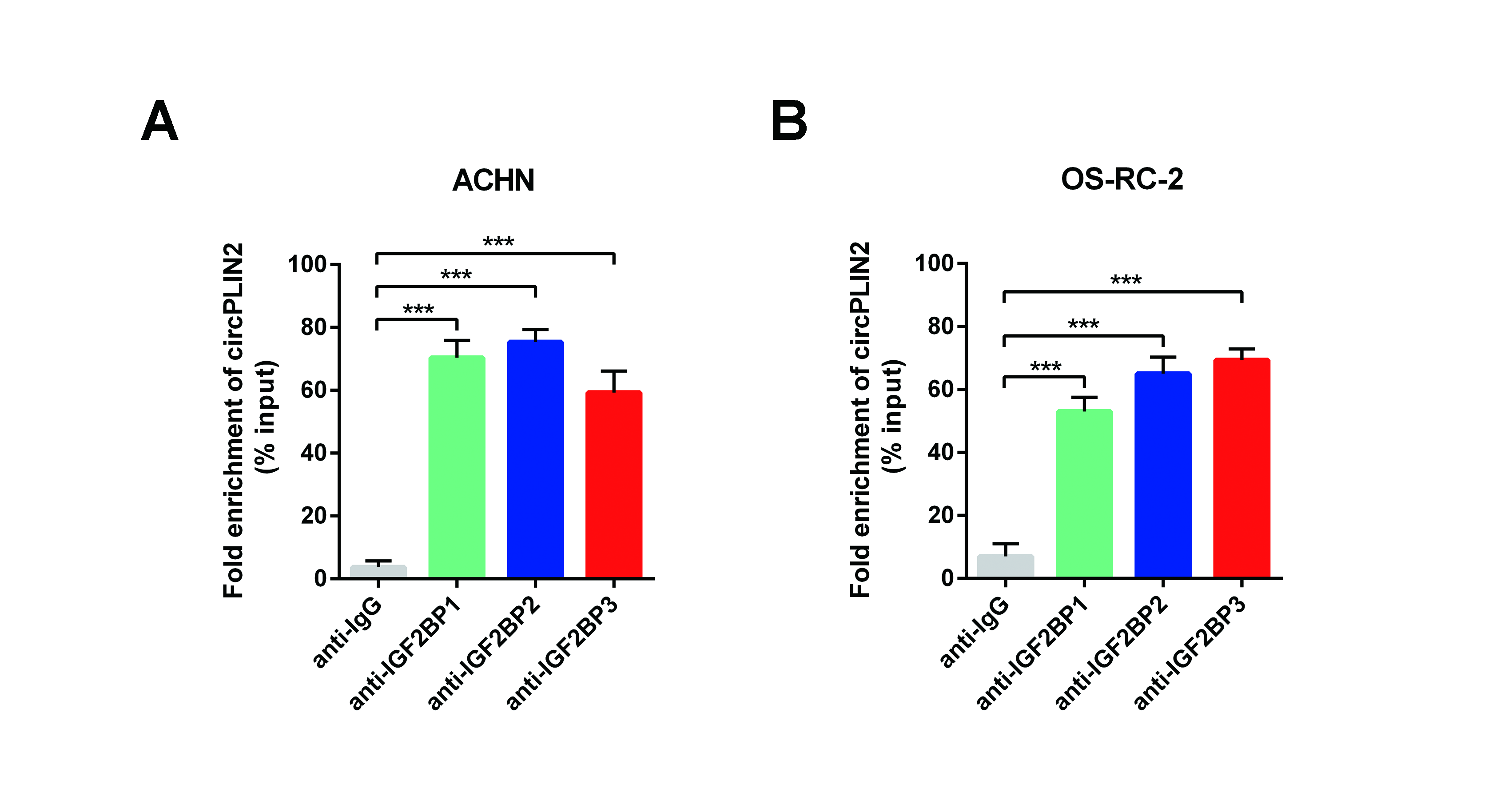

Supplement: Supplementary file 7 — Supplementary Figure 2 [file 41419_2022_5488_MOESM7_ESM.tif]

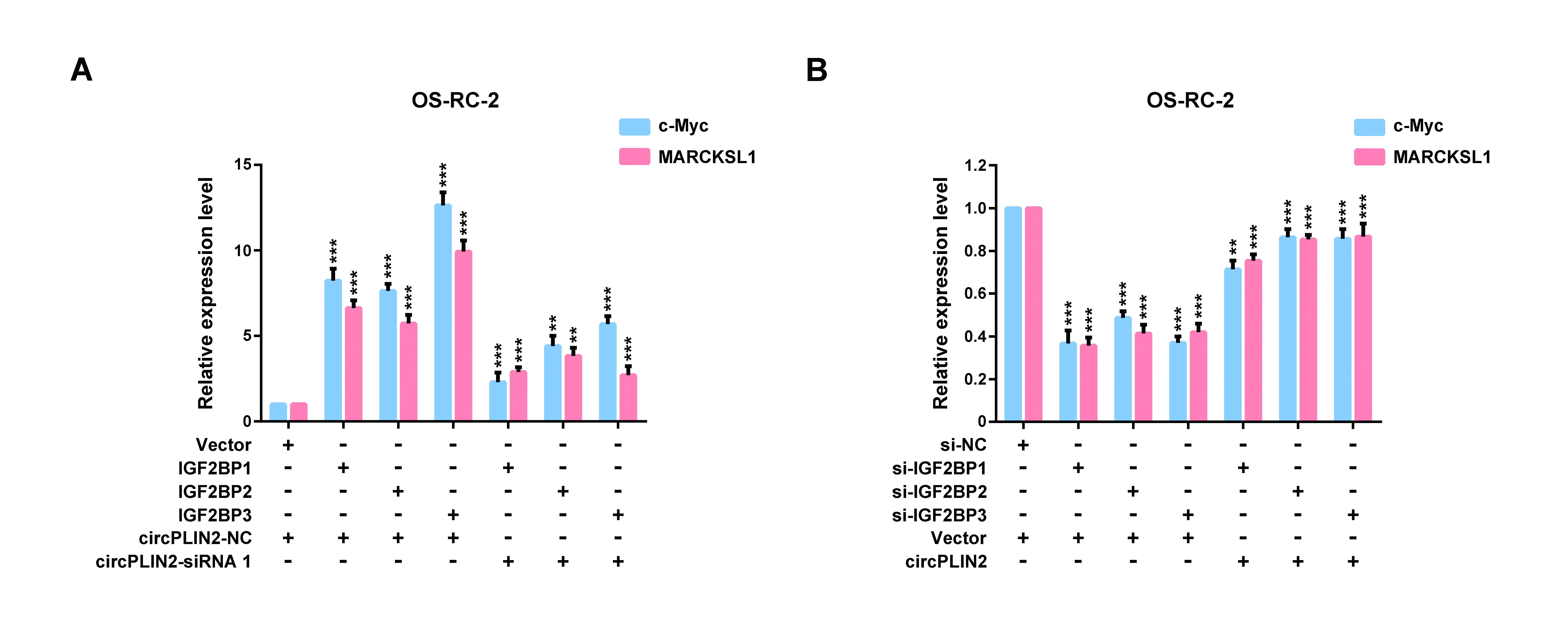

Supplement: Supplementary file 8 — Supplementary Figure 3 [file 41419_2022_5488_MOESM8_ESM.tif]

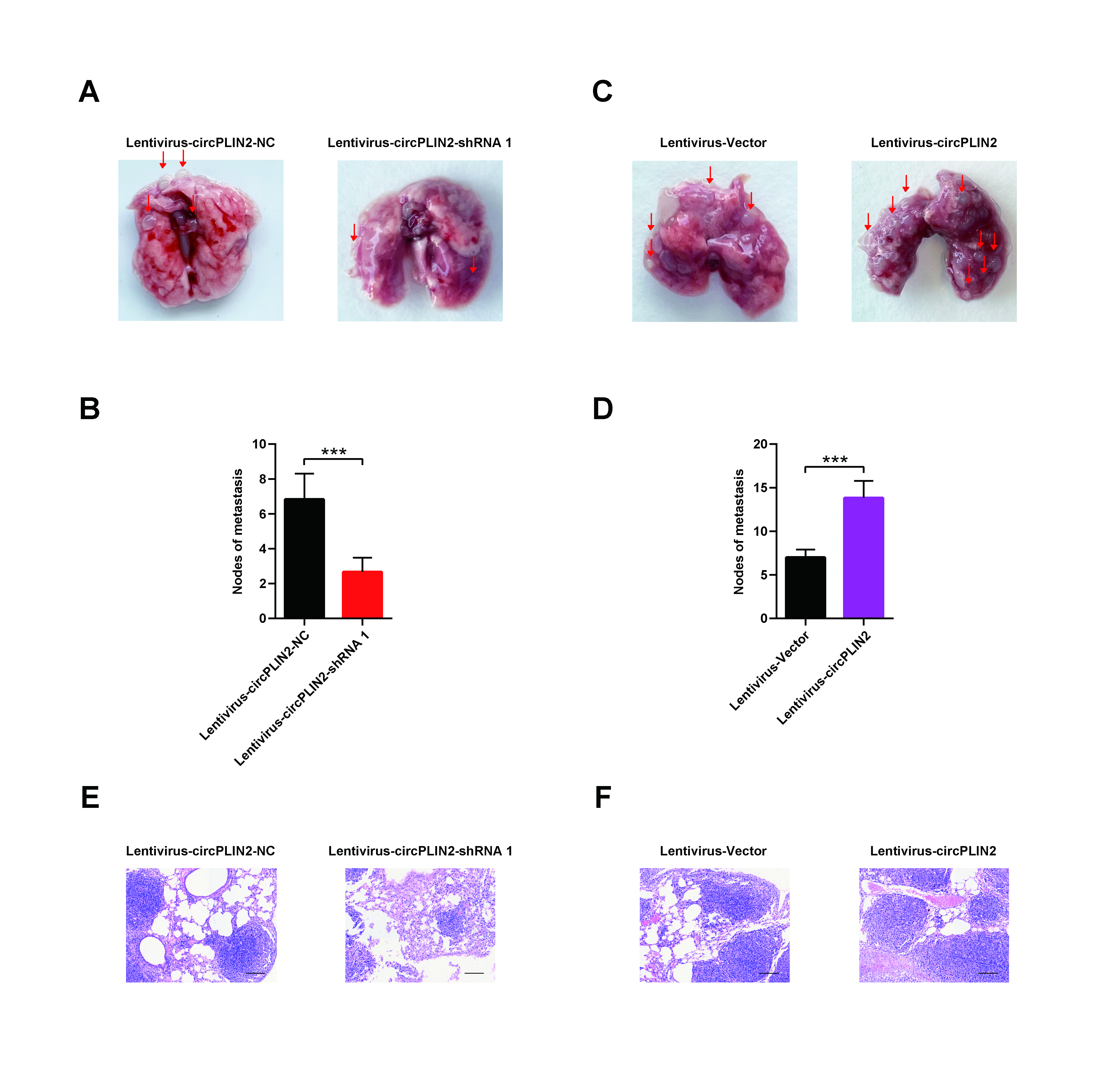

Supplement: Supplementary file 11 — Supplementary Figure 6 [file 41419_2022_5488_MOESM11_ESM.tif]
