# Supplementary material for: circPLIN2 promotes clear cell renal cell carcinoma progression by binding IGF2BP proteins and miR-199a-3p
Source: Cell Death Dis. 2022 Dec 9;13(12):1030. doi: 10.1038/s41419-022-05488-z (PMC9734136; doi:10.1038/s41419-022-05488-z)
Supplement: Supplementary file 12 — Supplementary Figure Legend [file 41419_2022_5488_MOESM12_ESM.docx]

**Supplementary Fig. 1 Raw western blot bands. A** Uncropped original western blots for Figure 4E.

**Supplementary Fig. 2 circPLIN2 binds to IGF2BP proteins in ccRCC cells. A-B** RNA immunoprecipitation analysis of the fold enrichment of circPLIN2 with anti-IGF2BP1 antibody, anti-IGF2BP2 antibody or anti-IGF2BP3 antibody in ACHN **(A)** and OS-RC-2 **(B)** cells. The anti-IgG group was used as the control. Two-tailed Student’s t test. The error bars represent S.D. ***p < 0.001.

**Supplementary Fig. 3 Knockdown or overexpression of circPLIN2 reverses the increases in the stability of the c-Myc and MARCKSL1 mRNAs induced by overexpression of IGF2BP proteins or decreases induced by knockdown of IGF2BP proteins.** **A** RT–qPCR analysis of the relative expression levels of c-Myc and MARCKSL1 in OS-RC-2 cells transfected with IGF2BPs or vector and circPLIN2-siRNA 1 or circPLIN2-NC. **B** RT–qPCR analysis of the relative expression levels of c-Myc and MARCKSL1 in OS-RC-2 cells transfected with si-IGF2BPs or si-NC and circPLIN2 or vector. Two-tailed Student’s t test. The error bars represent S.D. **p < 0.01 and ***p < 0.001.

**Supplementary Fig. 4 Neither knockdown nor overexpression of IGF2BP proteins alters the expression of circPLIN2.** **A-B** RT–qPCR analysis of the relative expression level of circPLIN2 in ACHN **(A)** and OS-RC-2 **(B)** cells transfected with si-IGF2BPs or si-NC. **C-D** RT–qPCR analysis of the relative expression level of circPLIN2 in ACHN **(C)** and OS-RC-2 **(D)** cells transfected with IGF2BPs or vector. Two-tailed Student’s t test. The error bars represent S.D. ns, not significant.

**Supplementary Fig. 5 Binding site information.** **A** circPLIN2 sponges miR-199a-3p and mutation of its binding site. **B** ZEB1 sponges miR-199a-3p and mutation of its binding site.

**Supplementary Fig. 6 circPLIN2 promotes ccRCC tumor metastasis in vivo. A** Representative images of lung metastasis in the lentivirus-circPLIN2-shRNA 1 and lentivirus-circPLIN2-NC groups. **B** The number of metastatic nodules in the lentivirus-circPLIN2-shRNA 1 and lentivirus-circPLIN2-NC groups. **C** Representative images of lung metastasis in the lentivirus-circPLIN2 and lentivirus-vector groups. **D** The number of metastatic nodules in the lentivirus-circPLIN2 and lentivirus-vector groups. **E** HE staining of metastatic nodules in the lungs from the lentivirus-circPLIN2-shRNA 1 and lentivirus-circPLIN2-NC groups. Scale bar, 100 μm. **F** HE staining of metastatic nodules in the lungs from the lentivirus-circPLIN2 and lentivirus-vector groups. Scale bar, 100 μm. Two-tailed Student’s t test. The error bars represent S.D. ***p < 0.001.
