# Supplementary material for: circPLIN2 promotes clear cell renal cell carcinoma progression by binding IGF2BP proteins and miR-199a-3p
Source: Cell Death Dis. 2022 Dec 9;13(12):1030. doi: 10.1038/s41419-022-05488-z (PMC9734136; doi:10.1038/s41419-022-05488-z)
Supplement: Supplementary file 13 — Certificate of English Language Editing [file 41419_2022_5488_MOESM13_ESM.pdf]

Editing Certificate

This document certifies that the manuscript

circPLIN2 promotes clear cell renal cell carcinoma progression by binding IGF2BP proteins and miR-199a-3p

prepared by the authors

Bin Zhao, Cong Huang, Jie Pan, Hao Hu, Xiaojuan Liu, Kaoyuan Zhang, Fenli Zhou, Xin Shi, Jun Wu, Bo Yu, Xiaofan Chen and Wei Zhang

was edited for proper English language, grammar, punctuation, spelling, and overall style by one or more of the highly qualified native English speaking editors at AJE.

This certificate was issued on **November 2, 2022** and may be verified on the AJE website using the verification code **7274-5EC5-606C-3CD6-DB15**.

Neither the research content nor the authors' intentions were altered in any way during the editing process. Documents receiving this certification should be English-ready for publication; however, the author has the ability to accept or reject our suggestions and changes. To verify the final AJE edited version, please visit our verification page at aje.com/certificate. If you have any questions or concerns about this edited document, please contact AJE at support@aje.com.

AJE provides a range of editing, translation, and manuscript services for researchers and publishers around the world. For more information about our company, services, and partner discounts, please visit aje.com.
